# Supplementary material for: Malaria protection due to sickle haemoglobin depends on parasite genotype
Source: Nature. 2021 Dec 9;602(7895):106–11. doi: 10.1038/s41586-021-04288-3 (PMC8810385; doi:10.1038/s41586-021-04288-3)
Supplement: Supplementary file 1 — This file contains Supplementary text, Figs. 1–6, Methods and references. [file 41586_2021_4288_MOESM1_ESM.pdf]

nature portfolio

https://doi.org/10.1038/s41586-021-04288-3

Supplementary information

Malaria protection due to sickle haemoglobin depends on parasite genotype

In the format provided by the authors and unedited

Malaria protection due to sickle haemoglobin depends on parasite genotype - supplementary figures, methods and text.

1. SUPPLEMENTARY FIGURES **2**

1.1. SUPPLEMENTARY FIGURE 1 2
1.2. SUPPLEMENTARY FIGURE 2 3
1.3. SUPPLEMENTARY FIGURE 3 4
1.4. SUPPLEMENTARY FIGURE 4 5
1.5. SUPPLEMENTARY FIGURE 5 6
1.6. SUPPLEMENTARY FIGURE 6 7

2. SUPPLEMENTARY METHODS **8**

2.1. BUILDING A COMBINED DATASET OF HUMAN AND *P.FALCIPARUM* GENOTYPES IN SEVERE CASES 8
2.1.1. Overview *8*
2.1.2. Parasitaemia measurements *8*
2.1.3. Sequencing using whole DNA samples from high-parasitaemia infections *8*
2.1.4. Sequencing using Selective Whole Genome Amplification (SWGA) *8*
2.1.5. P.falciparum genotype calling *9*
2.1.6. P.falciparum variant filtering *9*
2.1.7. Generating PfEBA175 'F' segment calls *9*
2.1.8. P.falciparum sample filtering *10*
2.1.9. Curation of joint human-Pf analysis datasets *10*
2.2. MODELLING ASSOCIATION OF HOST AND INFECTION GENOTYPES 11
2.2.1. Basic association model *11*
2.2.2. Interpretation when there is no within-host evolution *12*
2.2.3. Interpretation of the general model *12*
2.2.4. Possible causes of association *12*
2.3. ESTIMATION OF POPULATION RELATIVE RISKS USING MULTINOMIAL LOGISTIC REGRESSION 14
2.3.1. Estimation using a case-population sample *14*
2.4. IMPLEMENTING LOGISTIC REGRESSION TO TEST FOR HOST/PARASITE ASSOCIATION 16
2.4.1. Approximating the sampling distribution of the posterior mode *16*
2.4.2. Implementation using a log-F prior *18*
2.5. INTERPRETATION OF STATISTICAL EVIDENCE FOR HOST-PARASITE ASSOCIATION 18
2.5.1. Thresholds for interpretation of P-values *18*
2.5.2. Interpretation of Bayes factors *19*

3. SUPPLEMENTARY TEXT **20**

3.1. INVESTIGATION OF ADDITIONAL SIGNALS OF ASSOCIATION 20
3.1.1. Overall interpretation of additional signals *20*
3.1.2. Association between GCNT2 and two regions of the Pf genome *20*
3.1.3. Association between HLA alleles and variation in several regions of the Pf genome *20*
3.1.4. Association with ABO, ATP2B4 and glycophorin variation *21*
3.1.5. Additional associations with HbS *21*
3.1.6. Investigation of HbS-MSP1 association *21*
3.2. FUNCTIONAL INFORMATION ON THE *PFSA* LOCI 21
3.2.1. Relevant gene and protein identifiers *21*
3.2.2. Information on protein function *22*
3.2.3. Dispensability of HbS-associated genes *22*
3.2.4. Relationship of Pfsa1+ and Pfsa2+ alleles to PEXEL motifs *23*
3.2.5. Pfsa gene expression in 3D7 parasites *24*
3.2.6. Increased expression of PF3D7_1127000 in Pfsa+ parasites *24*
3.3. LINEAR MIXED-MODEL BASED ANALYSIS OF HBS-*PFSA* ASSOCIATION 25

4. SUPPLEMENTARY REFERENCES **28**

1. Supplementary Figures

1.1. Supplementary Figure 1

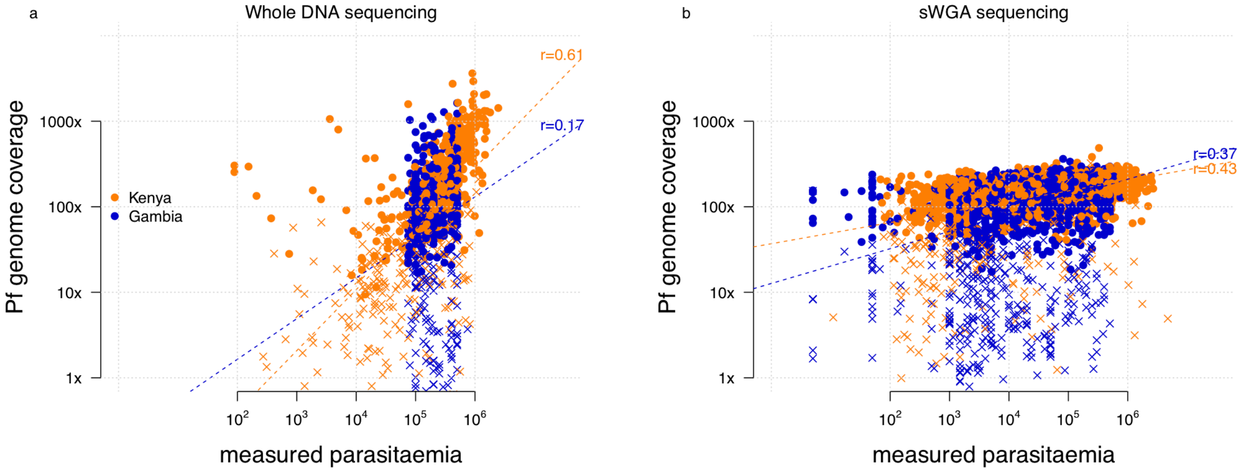

Supplementary Figure 1. *Pf* genome coverage compared to measured parasitaemia in whole DNA and SWGA sequencing pipelines.

For each sample (points) sequenced using the Whole DNA pipeline (panel a) or sWGA pipeline (panel b), figure shows the average per-base coverage of reads aligned to the Pf3D7 genome (y axis) against the P.falciparum parasitaemia (parasitised RBCs / ul blood) measured using blood slide at the time of ascertainment (x axis) in each country (colours). Crosses denote samples that failed QC metrics (detailed in **Supplementary Methods** and **Extended Data Figure 1**) and were excluded from our analysis dataset. Lines show linear regression fit of $\log_{10}$(coverage) against $\log_{10}$(parasitaemia), with labels indicating the estimated correlation.

1.2. Supplementary Figure 2

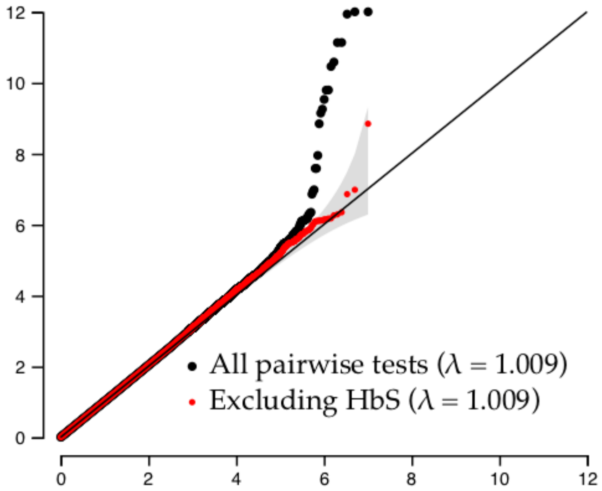

Supplementary Figure 2. Quantile-quantile plot for test of association between pairs of human and P.falciparum alleles

Plot shows observed -log10 P-value (y axis) against expection under the null model of no association (x axis) for tests of association between human and pf alleles. Tests are conducted using logistic regression across 3,346 samples, with the imputed human genotype as predictor and the parasite genotype as outcome. An indicator of country (Gambia or Kenya) was included as a covariate. Black (respectively red) points reflect quantile-quantile plot for all tests (black) or after excluding comparisons with HbS (red points), with the corresponding median lambda values shown in the legend. Grey area depicts the 99% confidence interval for the observed value, computed pointwise for each black point using the order statistics for a uniform distribution. Only comparisons where the minor allele count of the human variant for samples carrying either *Pf* genotype is at least 20 are shown (computed in expectation across the imputed genotype distribution, where relevant).

1.3. Supplementary Figure 3

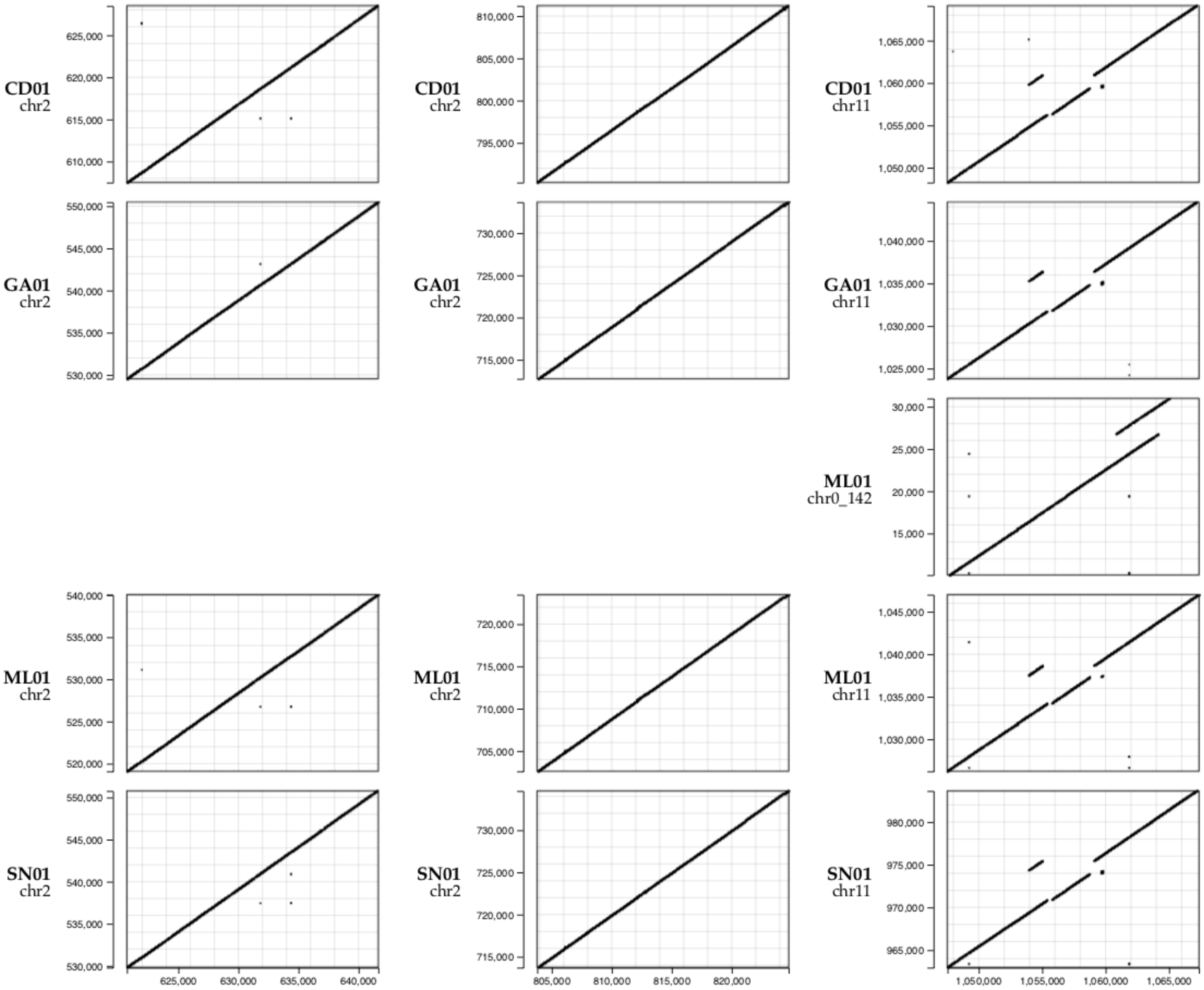

Supplementary Figure 3. K-mer sharing between Pf3D7 and *P.falciparum* genome assemblies that carry *Pfsa*+ alleles.

For each of a set of genomes of *Pf* isolates previously assembled from PacBio data [1] (rows), the plot shows short DNA sequences of length 50 (50-mers, black points) that are shared between the Pf3D7 reference genome (x axis) and the specified genome assembly (y axis). Points on the diagonal of each panel indicate similar DNA structure between the two assemblies, while off-diagonal points and breaks indicate potential structural variation. The *Pf* isolates selected are those that carry the *Pfsa*+ allele at at least one of the three lead HbS-associated SNPs in *Pfsa* regions. *Pfsa* genotypes were determined by aligning a 101-bp segment centred on each SNP in Pf3D7 to the corresponding assembly, and inspecting the relevant assembly base. In the notation of **Figure 2** the combined genotypes are: CD01 (Congo) : + + +; GA01 (Gabon): + - +; SN01 (Senegal): - - +; ML01 (Mali): - - +. Flanking sequence to the chr 11 locus aligned to two contigs in the ML01 assembly and both of these contigs are shown. ML01 was previously identified as containing a mixed infection[1]. Detail of the top-right panel can be seen in **Extended Data Figure 9**.

1.4. Supplementary Figure 4

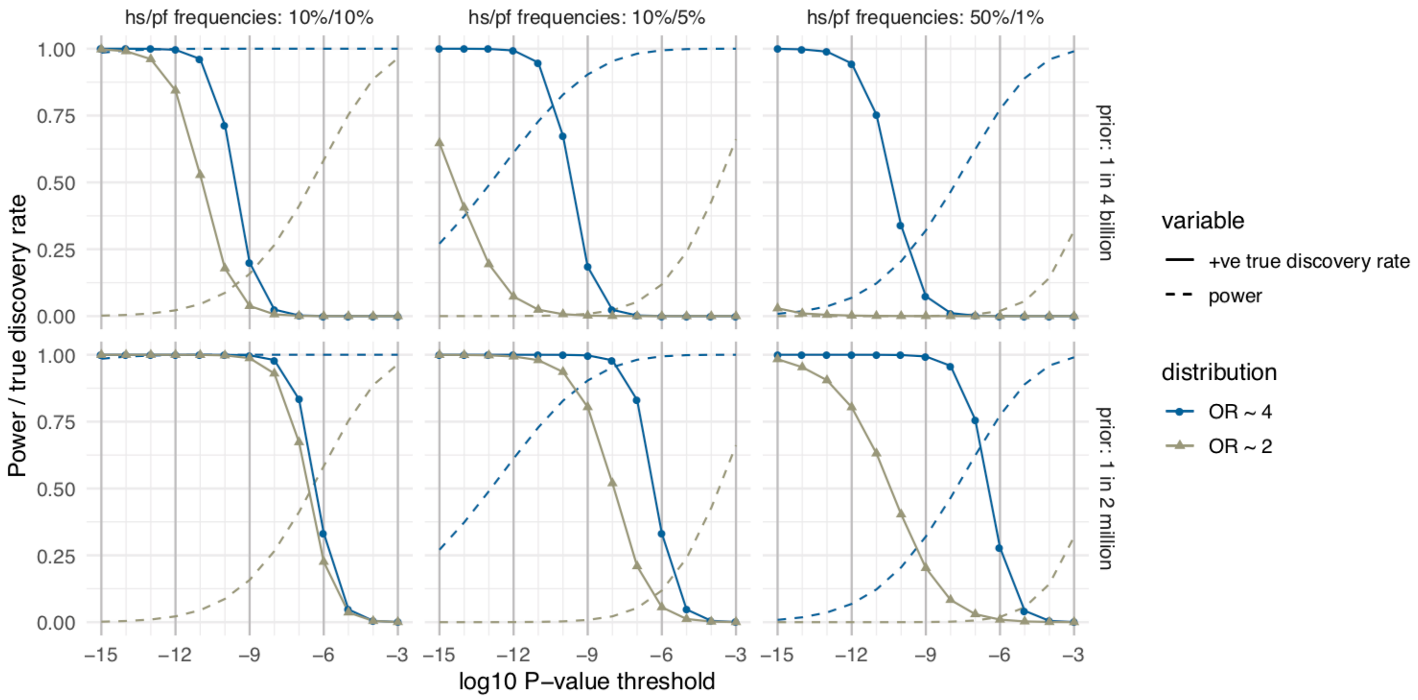

Supplementary Figure 4. Illustration of association test power and probability of association.

Plot shows approximate association test power (dashed lines) and probability of association (solid lines) for a range of P-value thresholds (x axis) under a range of scenarios (panels and line colours / point shapes). We assume a sample size of 3,346 to match our discovery analysis. The panels vary by prior probability of association (rows) and by the human and parasite variant frequencies (columns), while the line colour and point shape denotes the assumed association effect size as shown in the legend. The probability of association is computed as $P(\text{association}|p < T)$; the power is defined as as $P(p < T|\text{association})$, where T is the given threshold. Results are computed using an approximation to the association test standard error as described in **Supplementary Methods**.

1.5. Supplementary Figure 5

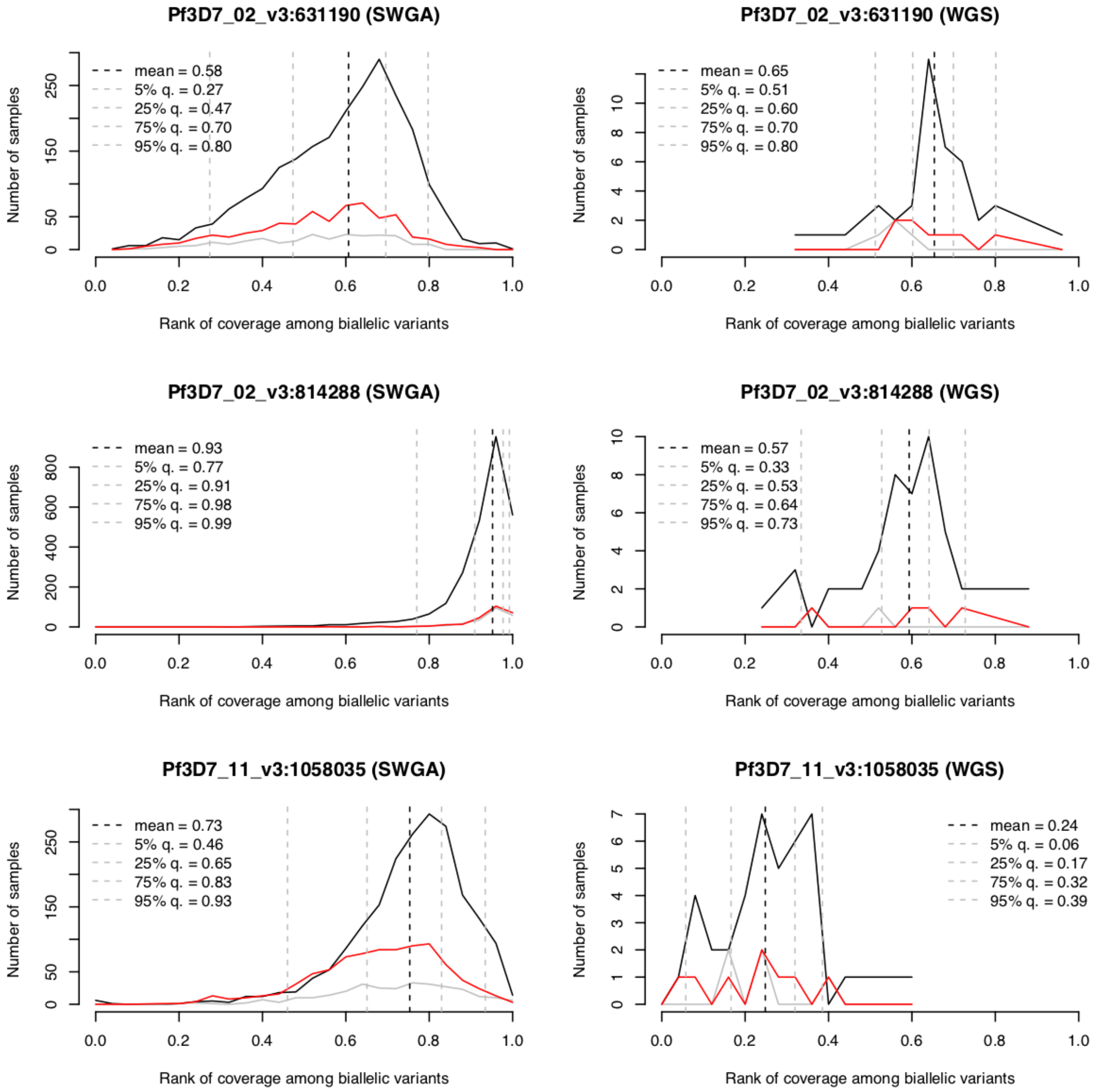

Supplementary Figure 5. Analysis of sequence read coverage at the *Pfsa* sites.

Panels shows sample counts (y axis) against normalised read coverage (x axis) at each of the *Pfsa1* (chr2:631,190), *Pfsa2* (chr2: 814,288) and *Pfsa3* (chr11:1,058,035) lead variants (rows), for both SWGA and whole DNA-sequenced samples (columns). For each sample the site read coverage was normalised by computing the rank of the per-site coverage among all biallelic sites called in our data. Results are separated by the allele carried at the focus SNP (black, reference allele; red, non-reference allele; grey, mixed genotype call). Vertical dashed lines show the mean and quantiles of the distribution of ranks as shown in the legend.

1.6. Supplementary Figure 6

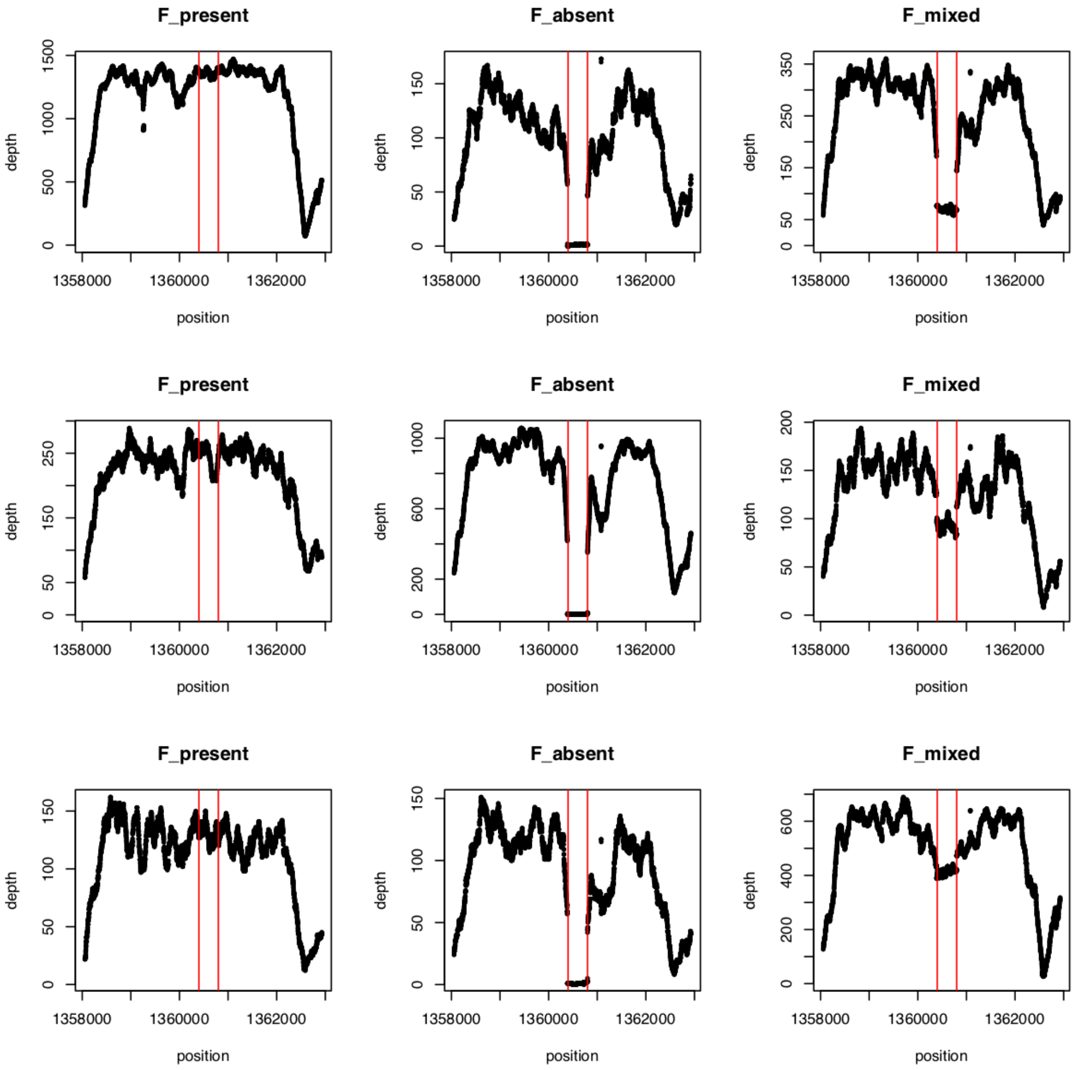

Supplementary Figure 6. Examples of EBA175 ‘F’ segment calling.

Panels show depth of reads aligned to the Pf3D7 genome at each site across the region of PfEBA175, for selected samples. The location of the ‘F’ segment (Pf3D7_07_v3:360,400-1,360,800) is shown between red vertical lines. The genotype called by our calling process (detailed in **Supplementary Methods**) is indicated in the panel label.

2. Supplementary Methods

2.1. Building a combined dataset of human and *P.falciparum* genotypes in severe cases

2.1.1. Overview

The following sections describe the sequencing and curation of a dataset of genome-wide *P.falciparum* (*Pf*) genetic variation and human genotypes from severe malaria cases collected in The Gambia and Kenya. A diagram of this process is presented in **Extended Data Figure 1**.

2.1.2. Parasitaemia measurements

Parasitaemia measurements were obtained for all case samples based on thin or thick blood slides at the time of sample ascertainment. Data were curated to produce a single parasitaemia measurement per individual, the count of red blood cells containing *P.falciparum* parasites per microlitre of blood (pRBCs/ul). Observed parasitaemia rates varied from very low (reported as < 10) to over 10^6 pRBCs/ul. which represents extremely high parasitaemia (hyperparasitaemia) given typical RBC blood count of 5×10^6 RBCs/ul.

Subsequent to selection of samples for whole DNA sequencing described below, we further linked Kenyan data to an updated set of parasitamia values re-curated from source measurements [2]. We use these values in **Supplementary Figure 1**.

2.1.3. Sequencing using whole DNA samples from high-parasitaemia infections

We assessed the scope for generating coverage of the *P.falciparum* genome from sequencing whole DNA as follows. We assumed an average quantity of 40 nanograms (ng) human DNA per ul blood (based on ~5,000 white cells / ul) and that a single parasite genome weighs on the order of 25×10^{-6} ng. A parasitaemia rate of $m \times 10^6$ pRBCs/ul thus corresponds to $25m$ ng/ul. This suggests sequencing whole DNA would yield a fraction of $\frac{25m}{25m+40}$ reads originating from the *Pf* genome. For parasitaemias on the order of $10^4 - 10^6$ pRBCs/ul we would therefore expect approximately 0.6% – 40% of reads would arise from the *Pf* genome. For approximately 100Gbp sequencing yield and a 23Mbp genome, this suggests between 25 to over 1,000-fold *Pf* genome coverage might be realizable using this method, depending on parasitaemia levels, although in practice we might expect this to reduce somewhat due to various forms of attrition.

Motivated by this calculation we selected a subset of case samples in each population from among those having the highest measured parasitaemia, targeting those having $>10^5$ pRBCs/ul. The Kenyan dataset sequenced also included a number of additional samples with lower parasitaemia measurements; these were selected based on their genotype at the human chromosome 4 glycophorin locus. All samples were sequenced on the Illumina XTEN platform at the Wellcome Sanger Institute. In total we obtained data for N=1,071 cases including 483 Gambians and 588 Kenyans. We aligned all reads to a combined human/parasite genome, obtained by concatenating the GRCh37 human genome reference assembly and version 3 of the Pf3D7 reference sequence [3] using BWA mem. Duplicate reads were marked with Picard MarkDuplicates and we extracted reads aligning to the *Pf* genome for downstream analysis.

To assess *Pf* sequencing performance we plotted coverage of the *Pf* genome from sequencing of whole DNA against measured parasitaemia (**Supplementary Figure 1**). Although coverage was strongly correlated with measured parasitaemia, this correspondence was incomplete, and we noted 2 Gambian case samples with estimated zero coverage, and a larger set of 66 Gambian and 118 Kenyan cases with < 10-fold coverage. Many of these samples had relatively high measured parasitaemia and substantial coverage of the human genome (e.g. 133 with measured pRBC/ul > 10,000 and estimated > 20-fold coverage of the human genome as assessed across a region of chromosome 4). We interpret this discrepancy as resulting from a combination of noise in parasitaemia measurements as well as in possible limits to the accuracy of the curation of parasitaemia measurement data.

2.1.4. Sequencing using Selective Whole Genome Amplification (SWGA)

To capture plasmodium genomes from lower-parasitaemia samples, we used Selective Whole Genome Amplification [4] (SWGA) to amplify *Pf* DNA from all cases included in our study (N=5,128 cases).

SWGA was performed as previously described[4] except that we added a higher quantity (40ng) of gDNA into the reaction to allow for the mixture of parasite and human DNA. The resulting libraries were sequenced across multiple lanes on the Illumina XTEN platform at the Wellcome Sanger Institute. Reads aligning to the human reference were removed, and remaining reads from multiple lanes were merged to create sample-level read files. Remaining reads were aligned to version 3 of the Pf3D7 reference sequence using BWA mem. Duplicate reads were marked with Picard MarkDuplicates.

We plotted coverage of the *Pf* genome from SWGA sequencing against measured parasitaemia (**Supplementary Figure 1**). We observed relatively high coverage of the Pf3D7 genome across all reported parasitaemia levels. Sequence coverage was however variable with e.g. approximately 5% of samples having especially low coverage (258 of 5190 samples with < 10-fold coverage).

2.1.5. *P.falciparum* genotype calling

To produce a robust set of parasite genotype calls we used an established pipeline that has previously been used to survey *P.falciparum* populations [5]. This pipeline uses GATK 4.0 HaplotypeCaller to identify genetic variants and to call genotypes jointly across all sequenced samples. Briefly, we first ran GATK HaplotypeCaller for each sample to generate a per-sample gVCF file, which represents a compressed view of the sequencing reads relevant for variant calling across all sites in the reference genome. We then used CombineGVCFs and GenotypeGVCFs to generate genotype calls across all samples. We specified a maximum of six alternate alleles in this process. SNPs and INDELs were then annotated using GATK's Variant Quality Score Recalibration (VQSR) based on a set of validated variants from crosses between *P. falciparum* laboratory strains [6] and based on genomic location.

Plasmodium parasites in humans have haploid genomes, but infections may consist of a mixture of parasite types due to co- or superinfection. The GATK pipeline described above calls variants as if genotypes were diploid. Heterozygous calls thus indicate regions for which reads containing both reference and non-reference alleles in substantial numbers are present (we refer to these as 'mixed' genotype calls), and homozygous calls represent variants for which the sample is largely unmixed. Although not perfect, this approach has been used previously as a practical way to handle genotype calls in mixed infections, and we adopted it here. Specifically, we treated mixed genotype calls as missing data in all analyses (except where noted), and treat the remaining homozygous calls as reflecting the true haploid genotype.

2.1.6. *P.falciparum* variant filtering

To pick a robust set of variants for analysis, we focussed on variants marked 'PASS' (i.e. with VQSLOD score > 0 and lying in the core genome which has been shown to be accessible to sequencing[6]). Inspection of remaining variants suggested the presence of many apparent multiallelic variants involving AT-rich sequence in the callset. Many of these are likely to reflect sequencing or calling errors, and we also excluded multiallelic variants from downstream analysis. In total, GATK called 4,974,562 variants across chromosomes 1-14, the apicoplast and mitochondrion, of which 2,793,802 were PASS and a subset of 1,716,459 were biallelic.

In addition to the variants above, we specifically included in our analysis variants in the region of *PfEBL1,* which is annotated as a pseudogene and lies in a subtelomeric region, but putatively encodes an erythrocyte invasion ligand in some *P.falciparum* species [7]. In total there were 1,339 biallelic variants with VQSLOD > 0 within the region Pf3D7_13_v3: 2,809,706-2,822,270.

2.1.7. Generating PfEBA175 'F' segment calls

In addition to the GATK-called variants described above, we also called a known deletion variant in *PfEBA175*, which encodes an invasion ligand that binds human Glycophorin A during merozoite invasion of erythrocytes[7]. *PfEBA175* is found in two forms, the 'F' type and 'C' types, which are distinguished by the presence of one of two non-overlapping ~400 bp DNA segments. The Pf3D7 reference genome carries the F segment located between positions 1,360,400 and 1,360,800 on chromosome 7. To identify the F segment in short-read sequence data, we computed sequence coverage across each base in these segments. We considered the F segment to be present if at least 350 of the 400 sites had a coverage >=5. We considered F absent if there was evidence for unusually low coverage across this region, defined as more than 350 of the 400 sites having coverage more than 2

standard deviations below the mean, as computed at nearby single-copy sequence. If both conditions were true, a mixed genotype call was assigned, and if neither were true a missing genotype was assigned. We note that this method directly assesses the presence of the F segment, but not of the C segment which is not present in the Pf3D7 reference; however, these segments are thought to rarely if ever cooccur[8].

2.1.8. *P.falciparum* sample filtering

To pick a robust set of samples for analysis, we filtered based on several criteria as follows. First, for samples multiply sequenced using the whole DNA and SWGA methods, we looked for discordance in genotype calls that would indicate sample mislabelling. No pair had > 2.5% discordance and we interpreted this as indicating that reads from corresponding SWGA and WGS read files represent the same DNA samples. Next, for whole DNA-sequenced samples with human genotyping available on the Illumina Omni 2.5M platform [9], we used VerifyBamId to confirm the identity of samples based on the human-aligned reads. We identified 25 samples with CHIPMIX > 2%, indicating sample contamination or sample mislabelling, and we excluded these samples (and corresponding SWGA samples) for downstream analysis. Third, we filtered samples based on the GATK v3.8.0 CallableLoci metric (defined as the proportion of reference bases where a sample has at least 5x coverage and such that at least 90% of covering reads have mapping quality >= 10, a criterion which is correlated with sequencing depth but is more directly relevant to variant calling). We excluded 758 samples where < 50% of reference bases were identified as callable by this metric, and a further 340 samples that had > 5% genotype missingness from downstream analysis. This process resulted in 4,440 samples from case individuals that passed filters.

2.1.9. Curation of joint human-*Pf* analysis datasets

For our main analysis we further restricted attention to two smaller sets of data based on the availability of human genotype data as follows. First, we formed a dataset consisting of the subset of 3,346 samples for which imputed human genotypes were previously analysed[9] ("the imputed dataset"). This contains 2,045 cases from The Gambia and 1,301 from Kenya, and contains no close relationships between human samples. We also formed a second dataset containing samples for which Sequenom MassARRAY genotyping was previously analysed[9] ("the sequenom dataset"). This set contains 4,083 samples (2,189 from The Gambia and 1,889 from Kenya) identified as not closely related based on the available genotypes[9] (although we note this determination is less certain than when using genome-wide genotype data). In total 3,246 samples were in both sets, leaving 825 with direct typing but no genome-wide data available.

2.2. Modelling association of host and infection genotypes

2.2.1. Basic association model

In **Methods** we interpret the odds ratio computed in severe malaria cases in terms of a simplified model of infection. Here we further describe this and extend to allow for the case where parasite genotypes might evolve through the course of an infection.

As in **Methods** we let A denote a population of susceptible individuals. We assume that we are interested in a specified set of parasite variants such that parasites have one of $J+1$ possible combined genotypes, denoted $j = 0, \cdots, J$. For a given individual we write $I = x$ or more briefly I_x to denote that the individual was bitten and infected with genotype x; more generally x might denote a mixture of parasite genotypes. For clarity, we define "infected" here to mean that parasites are injected into the bloodstream during a bite by an infected mosquito; infections might or might not successfully further invade host cells and continue to grow. Separately, we write $G = y$ to denote that the infection genotype at the time of measurement (i.e. sample ascertainment in our study) was y.

In principle, the infection-time (I) and ascertainment-time (G) genotypes might differ. This could happen if the initial infection is mixed and genetic drift or selection acts on parasites within-host, or if parasites mutate during the course of infection.

We write D to denote "individual has severe disease", and write D_y for "individual has severe disease with parasite genotype y" (i.e. $D_y = (D, G = y)$). Lastly, $E=e$ denotes a particular level of a host genotype. The following table summarises this notation.

Model notation	
A	Study population
D	Individual has severe malaria
D_y	Individual has severe malaria with parasite genotype y
$I = x$ or I_x	Individual was infected with parasite genotype x
$G = y$	Denotes parasite genotype at time of sampling
$E = e$	Individual has host genotype e

All the probabilities we discuss are conditional on the assumed population A, which we drop from the notation where convenient. The odds ratio for a particular genotype $G = y$ and host genotype $E = e$ computed in severe malaria cases, relative to baseline genotypes, can now be written as

$$OR_{G=y,E=e} = \frac{P(D_y, E = e|D)}{P(D_0, E = e|D)} \Big/ \frac{P(D_y, E = 0|D)}{P(D_0, E = 0|D)} \tag{S1}$$

The observed parasite genotype depends on the infection genotype, which is unobserved, so we must sum over it:

$$\begin{aligned} P(D_y, E = e|D) &= \sum_x P(D_y, I_x, E = e|D) \\ &= \frac{1}{P(D|A)} \cdot \sum_x P(D_y|I_x, E = e) P(I_x, E = e) \end{aligned} \tag{S2}$$

The first term in (S2) is independent of the genotypes and cancels out when forming the ratio (S1). Thus (S1) expands to

$$OR_{G=y,E=e} = \frac{\left(\frac{\sum_x P(D_y|I_x, E = e) P(I_x, E = e)}{\sum_x P(D_0|I_x, E = e) P(I_x, E = e)} \right)}{\left(\frac{\sum_x P(D_y|I_x, E = 0) P(I_x, E = 0)}{\sum_x P(D_0|I_x, E = 0) P(I_x, E = 0)} \right)} \tag{S3}$$

2.2.2. Interpretation when there is no within-host evolution

In **Methods** we make the simplifying assumption that $y = x$, that is, that genotypes do not change during the course of an infection. This is likely to be a broadly appropriate assumption for infections that are not initially mixed at the set of parasite variants of interest, since within-host mutation of specific bases is likely to be relatively rare [10] (at least until parasitaemia levels become high). In this case (S3) simplifies to

$$OR_{G=y,E=e} = \left(\frac{P(D|I_y, E = e)}{P(D|I_0, E = e)} / \frac{P(D|I_y, E = 0)}{P(D|I_0, E = 0)} \right) \times OR^{\text{biting}} \quad \text{(S4)}$$

where

$$OR^{\text{biting}} = \frac{P(I_y, E = e)}{P(I_0, E = e)} / \frac{P(I_y, E = 0)}{P(I_0, E = 0)} \quad \text{(S5)}$$

Equation (S4) is equivalent to expression (2) described in **Methods**. Since $y=x$ is the genotype at time of initial infection, $OR^{\text{biting}} = 1$ (for all genotypes e and y) is equivalent to statistical independence of host and parasite genotype at the time of infection. Further, if $OR^{\text{biting}} \equiv 1$, it can be shown that the first term in (S4) is one (for all genotypes e and y) if and only if host and parasite genotypes contribute multiplicatively to disease risk,

$$P(D|I_y, E = e) = \mu \times \frac{P(D|I_y)}{P(D|I_0)} \times \frac{P(D|E = e)}{P(D|E = 0)} \quad \text{(S6)}$$

where $\mu = P(D|I_0, E = 0)$ is the risk given baseline genotypes. Hence, $OR_{G=y,E=e} \neq 1$ implies either nonindependence of host and parasite genotypes at infection time or that host and parasite genotypes do not contribute multiplicatively to disease risk.

2.2.3. Interpretation of the general model

The general form of (S3) allows for within-host evolution and is more complex, but we show that the analogous behaviour holds: namely, $OR_{G=y,E=e} \neq 1$ implies either nonindependence between host and infection-time genotypes, or that host and parasite genotypes do not contribute multiplicatively to disease risk, in the sense that

$$P(D_y|I_x, E = e) = \mu \times \frac{P(D_y|I_x)}{P(D_y|I_0)} \times \frac{P(D_y|E = e)}{P(D_y|E = 0)} \quad \text{(S7)}$$

does not hold, where the expressions are now extended to express the risk of disease with a specific parasite genotype y given host and (possibly different) infection genotypes. To see this, note that neither μ, $P(D_y|I_0)$, nor the last ratio in (S7) depend on the infection genotype x. If (S7) holds they therefore cancel out of expression (S3). If host and parasite genotypes are independent at the time of biting then also $P(I_x, E = e) = P(I_x)P(E = e)$, leading to additional cancellation, so that

$$OR_{G=y,E=e} = \frac{\dfrac{\sum_x P(D_y|I_x)P(I_x)}{\sum_x P(D_0|I_x)P(I_x)}}{\dfrac{\sum_x P(D_y|I_x)P(I_x)}{\sum_x P(D_0|I_x)P(I_x)}} = 1$$

Thus, if we rule out nonindependence at time of biting, $OR_{G=y,E=e} \neq 1$ implies deviation from the multiplicative model (S7).

2.2.4. Possible causes of association

There are several mechanisms by which statistical nonindependence between host and observed parasite genotypes could arise in principle, and these make different predictions that can potentially be

tested. To illustrate the distinction between these mechanisms, we consider a simplified model of infection that separates out an initial phase of within-host evolution (that produces an observable level of parasitaemia and the observable genotype *G*) and a subsequent phase of infection in which disease status is determined (but parasitaemia and genotypes do not change). As above, an infection may be mixed and if so we interpret *G* to indicate the overall proportions of genotypes making up the infection. This model can be summarized in the following diagram which shows possible causal links between variables [11]:

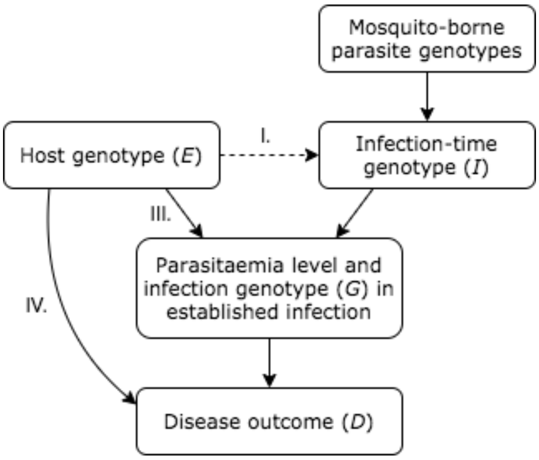

For the purposes of this illustration we ignore possible confounding factors not shown in the diagram. In this diagram there are several ways in which the odds ratio (S1) might deviate from unity as we detail below.

I. *Association induced by biting effects.* The dashed line in the diagram shows a possible influence of host genotype on parasite genotype at the time of infection. This would only seem to be possible if genotypes of possible hosts and mosquito-borne sporozoite genotypes can detected by infectious mosquitos (or the parasites they carry). (However, confounding factors, such as host and parasite population structure covariant with geography, could conceivably generate association along the same path as I.)

II. *Association induced by phenotyping.* In the diagram above, the disease status *D* is a collider [11] (i.e. is jointly determined by both host and parasite genotypes), and consequently conditioning on *D* could generate correlation between *E* and *I* even in the absence of a specific molecular or biological interaction. A well-known form of this is known as Berkson's paradox[12]; translating this to our setting, this could occur if the clinically determined criteria for severe malaria arise in infections of individuals who do not carry protective genotypes, or in infections with pathogenicity-causing alleles, or both. Specifically this leads to association when the contributions of host and parasites alleles to disease risk do not follow the multiplicative model (S6).

To assess this in relation to the HbS association described in main text, we investigated whether each of the HbS-associated alleles was also associated with other known host protective alleles. Specifically we considered the protective homozygous AA genotype at rs4951377 [13,14]; genotypes carrying the G allele at rs186873296 (which tags the Dantu blood group variant DUP4) under an additive model [15,16]; and O blood group encoded by rs8176719[14]. We observed little evidence for association (P > 0.05 for all nine tests; P-values were not significantly divergent from a uniform distribution using a Kolmogorov-Smirnov test) although we noted that all but one estimated effect size was positive. The strongest estimated association was for rs186873296 and *Pf* chr11:1,058,035 (*OR* = 1.38; 95% CI 0.99-1.94); this estimate reduced somewhat after additionally including HbS as a predictor.

We also noted that the HbS-associated alleles are observed at higher frequency in community samples [5] than in the severe cases studied here (with one exception for the

chr2:814,288 T allele in Gambia, which is not present in the community-sampled data but is at ~1.5% frequency in our sample of severe cases; **Figure 3**). These data do not support a formal statistical comparison due to differences in sampling, but do not appear to indicate a strong overall pathogenicity effect of the three HbS-associated alleles. Thus, although out data do not formally rule this out, neither of these comparisons appears to support a nonspecific effect in which host protective alleles and parasite pathogenicity alleles become correlated purely due to the definition of severe disease.

III. *Host genotype effects on within-host parasite genotypes*. The most plausible explanation for association between host and parasite genotypes may be that host genotypes affect the within-host fitness of parasites, in a way that varies with parasite genotype. For the HbS effect described in main text, this would occur if parasites with the *Pfsa*+ alleles are better adapted than other parasites to growing and infecting erythrocytes of individuals with HbS genotype, compatible with the counts observed in **Figure 2**. In the simplified model above this would lead to systematic association of host and parasite genotypes in all infections (regardless of symptom severity), but we note that in real settings this would depend on the strength and timing of selection within the course of disease. The appearance of association in uncomplicated cases from Mali (**Supplementary Table 2**) may support this interpretation. Within-host selection of this type would likely also lead to effects on transmission of parasite genotype, and thus potentially place selection pressure on parasite populations.

IV. *Interactions determining disease tolerance.* In principle another possibility (separate in the above simplified model) is that host and parasite genotypes jointly determine host tolerance to infection, without otherwise affecting parasite development. Host and parasite genotypes would then appear associated in a sample of severe cases. In the simplified model above, an effect of this type would be unobservable in asymptomatic cases since the effect is specific to severe disease phenotype.

2.3. Estimation of population relative risks using multinomial logistic regression

2.3.1. Estimation using a case-population sample

We consider estimating the relative risk for severe disease observed with a particular parasite genotype y in population A,

$$RR_{E=e}(y) = \frac{P(D_y|E = e, A)}{P(D_y|E = 0, A)} \tag{S8}$$

Here, as above $E=e$ denotes a particular host genotype (e.g. HbS genotype in **Figure 2**) and the relative risk is measured with respect to a chosen baseline genotype $E=0$ (i.e. non-HbS genotypes in **Figure 2**). Application of Bayes' theorem to (S8) shows that

$$RR_{E=e}(y) = \frac{P(E = e|D_y)}{P(E = 0|D_y)} \Big/ \frac{P(E = e|A)}{P(E = 0|A)} \tag{S9}$$

Expression (S9) can be recognized as an odds ratio, specifically the odds ratio comparing the frequency of the exposure $E=e$ in disease cases with genotype y relative to the general population. It can therefore be estimated from a sample of disease cases and population controls. More generally, we show the following:

Lemma. *Suppose a disease with J+1 possible types* $y = 0, \cdots, J$ *follows a linear log-risk model in the population A,*

$$\log P(D_y|E = e, Z = z, A) = \beta e + z^t \gamma \tag{S10}$$

where Z denotes a vector of covariates, and β *and* γ *are log-relative risks for the exposure e and the covariates respectively. Assume for simplicity that Z consists of a single categorical covariate (i.e. z is*

a vector of zeros and ones with exactly one entry equal to 1). Suppose S is a case-population sample in which sampling is independent of host and parasite genotype, given the disease status and covariates. Then in the sample the multinomial logistic regression model holds:

$$\log \frac{P(D_y|E = e, Z = z, S)}{P(D_-|E = e, Z = z, S)} = \beta e + z^t \gamma' \tag{S10}$$

where D- indicates that an individual was sampled as a population control, and the coefficient β of the host genotype e is the same as in the full population model.

Note. This lemma is a counterpart of the well-known result that if a logistic regression model holds for a disease in the general population, then a transformed logistic regression model holds in a sample of disease cases and strict (non-diseased) controls [17]. We apply Lemma 1 in **Figure 2** to estimate the relative risk conferred by HbS on disease across multiple parasite genotypes.

Proof of lemma. Let Ω be the odds-ratio for disease of genotype *y* relative to the population,

$$\Omega = \frac{P(D_y|E = e, Z = z, S)}{P(D_-|E = e, Z = z, S)}$$

Applying Bayes theorem to numerator and denominator gives

$$\Omega = \frac{P(E = e|D_y, Z = z, S)}{P(E = e|D_-, Z = z, S)} \cdot K_y(z) \tag{S11}$$

where $K_y(z) = \frac{P(D_y|Z=z,S)}{P(D_0|Z=z,S)}$ is the ratio of cases of type *y* to controls in the study. Conditional independence of sampling on the genotypes further implies that we may replace S by A in the right hand side of (S11), giving

$$\Omega = \frac{P(E = e|D_y, Z = z, A)}{P(E = e|Z = z, A)} \cdot K_y(z)$$

Applying Bayes' theorem a second time to rewrite in terms of disease risk now gives

$$\Omega = P(D_y|E = e, Z = z, A) \cdot \frac{K_y(z)}{\kappa_y(z)}$$

where $\kappa_y(z) = P(D_y|Z = z, A)$ is the prevalence of disease type y in the population having the given covariate levels *x*.

By assumption

$$\begin{aligned} \log \Omega &= \log P(D_y|E = e, Z = z, A) + \log\left(\frac{K_y(z)}{\kappa_y(z)}\right) \\ &= \beta e + z^t \gamma + \log\left(\frac{K_y(z)}{\kappa_y(z)}\right) \\ &= \beta e + z^t \gamma' \end{aligned}$$

for the transformed parameter γ' defined as

$$\gamma' = \gamma + \begin{pmatrix} \log(K_y(z_1)/\kappa_y(z)) \\ \vdots \\ \log(K_y(z_d)/\kappa_y(z_d)) \end{pmatrix}$$

where $z_1, \cdots, z_d$ denote the d possible levels of the 0-1 covariate vector *z*.

2.4. Implementing logistic regression to test for host/parasite association

In main text and **Methods** we describe the use of logistic regression to estimate association between host genotypes (included as predictor variables) and parasite genotypes (included as outcome variables). Results for our discovery phase are summarized in **Figure 1**.

Logistic regression is susceptible to finite sample bias that leads to overestimation of effect sizes in a way which varies with the frequency of predictor and outcome variables. Given that comparisons between human and parasite variants at widely varying frequencies are being tested, we chose to mitigate this by fitting the model after including a regularizing prior distribution. Following previous recommendations [18] we chose a log-F(2,2) prior for this. The log-F(2,2) distribution is depicted below in comparison to a Gaussian(0, 1.87^2) distribution (for which 95% of the mass is concentrated on a similar interval centred at zero):

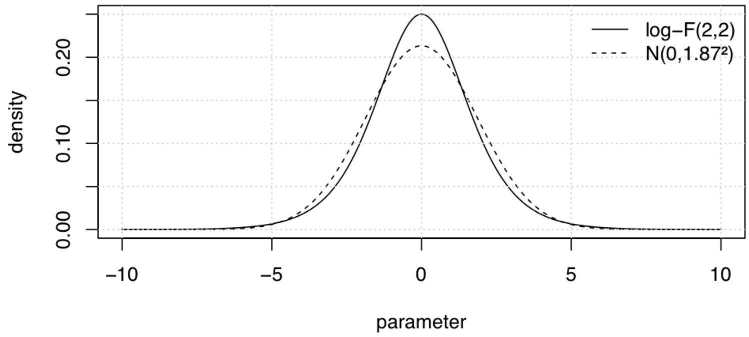

The log-F(2,2) distribution is slightly more concentrated near zero than the Gaussian (i.e. it provides greater regularization near zero) but has flatter tails (i.e. provides less regularization for parameter estimates that are far from zero). This captures an intuitive idea that most effects between individual host and parasite variants are likely to be small, but some large effects may exist.

The use of a prior enables computation of a Bayes factor for association at each pair of variants; we compute this using a Laplace approximation. A nonstandard calculation is also needed to compute a corresponding P-value; we do this by approximating the sampling distribution of the posterior mode as described in subsequent sections.

2.4.1. Approximating the sampling distribution of the posterior mode

We first review a standard approach that is often applied to (unregularized) regression. As sample sizes grow large the likelihood function is assumed to approach a Gaussian distribution near its maximum v (up to a scaling constant which does not depend on the parameter and is ignored below),

$$P(\text{data}|\theta) \approx N(\theta|v, V) \qquad \text{(S12)}$$

Here θ denotes the vector of parameters and V is a variance-covariance matrix expressing how sharply peaked the likelihood function is around its maximum. V can be computed as the inverse of minus the second derivative of the log-likelihood, evaluated at v.

As a function of the sample, the maximum likelihood estimate itself is assumed to become approximately normally distributed around the true parameter value θ_0 as sample sizes grow,

$$v \sim N\left(\theta_0, \frac{I}{n}\right) \qquad \text{(S13)}$$

Here I is another variance-covariance matrix (the Fisher information) that does not depend on the particular dataset being analysed, and n is the sample size. The factor of n in the covariance captures the fact that increasing sample size leads to estimates of increased precision, with standard errors approximately scaling as $1/\sqrt{n}$.

The standard theory links (S12) and (S13) by showing that (asymptotically as sample sizes grow large) the matrix V (which is estimated from the data) becomes approximately equal to $\frac{I}{n}$ (which is independent of the data) thus providing an effective way to compute P-values. Specifically, a "Wald test" P-value can be computed from the approximation $v \sim N(0, V)$ under the null model $\theta_0 = 0$; for a single component v_i of v this leads to

$$\text{P-value} \approx 2 \times F(-|v_i|; 0, V_i) \tag{S14}$$

where F is the cumulative distribution function of a Gaussian with the given mean and variance. (The use of $-|v_i|$ and the factor of 2 in this expression ensure that this computes a two-tailed P-value, i.e. the total mass under both tails of the distribution of parameter values greater or equal in magnitude to v_i).

We now consider computing P-values under regression regularized by a prior – we first consider a Gaussian prior with zero mean, i.e. defined as $\theta \sim N(0, \Sigma)$ for some variance-covariance matrix Σ. To do this, we use the same approximations as above to compute the approximate sampling distribution of the posterior mode. A Gaussian prior is particularly simple to use here because of the following well-known result which reflects the fact that the product of two gaussian densities is another gaussian density:

Lemma. *Under the approximation (S12), the posterior distribution is also Gaussian. Specifically*,

$$P(\theta|\text{data}) \approx N(\theta|\omega, \Omega) \tag{S15}$$

where $\Omega = (V^{-1} + \Sigma^{-1})^{-1}$ *and* $\omega = \Omega V^{-1} v$. *Under the approximation (S13), the posterior mode ω is therefore approximately distributed as*

$$\omega \sim N(\Omega V^{-1}\theta_0, \Omega V^{-1}\Omega) \tag{S16}$$

(The normalizing constant in (S15) can also be computed as another Gaussian function, which leads to the well-known computation of the approximate or asymptotic Bayes factor [19].)

It is instructive to consider these formulae in the case of a one-dimensional parameter and assuming $\theta_0 = 0$. In this case the matrices V and Σ are scalars and we have the simplifications:

$$\begin{aligned} \text{posterior mode } \omega &= \frac{\Sigma}{V + \Sigma} v \\ \text{posterior variance-covariance } \Omega &= V \cdot \left(\frac{\Sigma}{V + \Sigma}\right) \\ \text{sampling variance of } \omega &= V \cdot \left(\frac{\Sigma^2}{(V + \Sigma)^2}\right) \end{aligned} \tag{S16}$$

Thus the posterior mode ω is closer to zero than the maximum likelihood estimate v (i.e. it is a "shrinkage estimate"); the posterior variance Ω is smaller than the likelihood variance V; and the sampling variance of ω is smaller still. The degree of shrinkage in each case depends on the relative magnitude of the likelihood variance V and the prior variance Σ, with the extremes being $\Sigma = \infty$ (which produces no shrinkage at all) and $\Sigma = 0$ (which makes all three expressions equal to zero).

In our implementation, for each parasite variant and each human variant considered, we obtain the posterior mode ω by numerical approximation using a modified Newton-Raphson with line search [20]. We then compute the approximate posterior variance-covariance matrix $\widehat{\Omega}$ (computed as the inverse of negative the second derivative of the log-posterior at ω) and an approximate likelihood covariance $\widehat{V}$ (computed as the inverse of negative the second derivative of the log-likelihood at ω). By analogy with (S14) we then compute a P-value for the parameter of interest ω_i as

$$\text{P-value} = 2 \times F\left(-|\omega_i|; 0, \left[\widehat{\Omega}\widehat{V}^{-1}\widehat{\Omega}\right]_i\right) \tag{S17}$$

2.4.2. Implementation using a log-F prior

As described above, in our implementation we chose to use the log-F distribution which is a natural choice for logistic regression problems[18] and provides slightly stronger regularization near zero than a Gaussian with similar tails. There are therefore two ways in which (S17) might fail to give an accurate P-value. First, if the asymptotic approximations (S12) and (S13) fail then (S14) may not be accurate; in this case (S17) and (S14) might also differ. Second, the expressions might differ because of differences between the log-F prior and the Gaussian. To assess the impact of these, we conducted a simulation study as follows

1. We considered human and parasite variants at population frequencies of 1%, 5%, 10%, 20%, 30% and 50%.
2. For each frequency *f*, we simulated genotypes for 10 human variants in *N=3,346* samples by binomial sampling given the frequency.
3. For each frequency *f*, we also simulated genotypes for 10,000 parasite variants in *N=3,346* samples by binomial sampling given the frequency (thus representing no association between human and parasite genotypes).
4. We ran hptest to test for association between each human and parasite variant (3.6 million tests in total) with no prior applied and computed a Wald test P-value (S14) and a likelihood ratio test P-value.
5. We ran hptest a second time applying the log-F(2,2) prior and applied (S17) to recompute the P-value.
6. We plotted results stratified by the minimum count observed across all combined host and parasite genotypes at the two variants (i.e. the minimum value in the 2x2 contigency table formed by the two genotypes).

The following image shows a comparison of Wald test P-values and parameter estimates for the unregularized and log-F(2,2)-regularised regression.

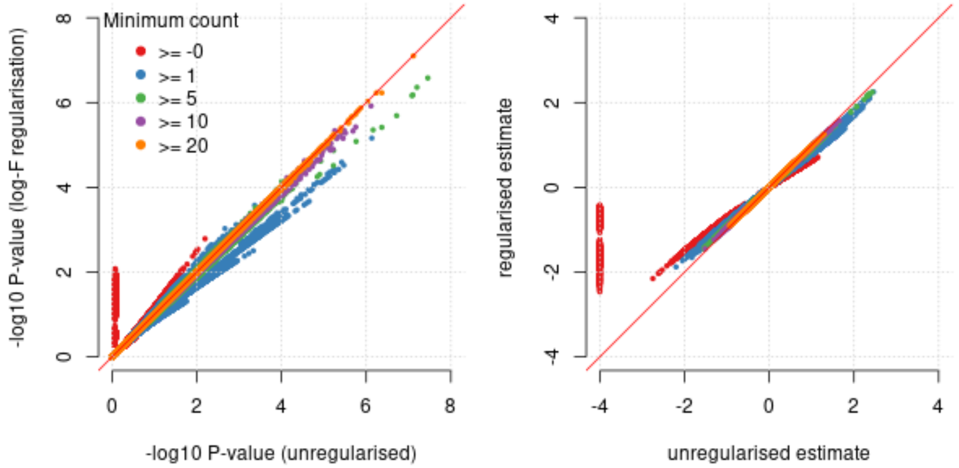

We noted that when the minimum combined genotype count is at least 20, the P-values computed by (S14) and (S17) are essentially identical, although discrepancies can be observed for lower counts; the P-value computed from regularised regression is typically more conservative in these cases. These discrepancies are similar in magnitude to those observed between Wald and likelihood ratio test P-values computed from unregularized regression. For smaller counts, inclusion of the prior has the desirable property that it generates less overestimation of effect size magnitude, including for some pairs of variants that generate extremely large estimates when the prior is not included.

2.5. Interpretation of statistical evidence for host-parasite association

2.5.1. Thresholds for interpretation of P-values

Consider possible association between a given host variant v and a parasite variant *w*. Let *A* stand for the statement "*v* and *w* are genuinely associated in severe malaria cases", and let ! *A* stand for the

converse statement (i.e. that the variants are unassociated). Let p denote the P-value for the test of association. For any chosen threshold value *T*, the evidence for association given a p-value < T can be derived as:

$$P(A|p < T) = \frac{P(p < T|A)P(A)}{P(p < T|A)P(A) + P(p < T|!A)P(!A)}$$

This expression can be simplified by noting that:

- The distribution of P-values for unassociated variants is uniform, i.e. $P(p < T|!A) = T$;
- The distribution of P-values for associated variants is reflected in the term $P(p < T|A)$, which is the association test power. The power in turn depends on the magnitude of the true effect and the variant frequencies and is investigated further below.
- The left-hand side of the formula is the posterior probability of association given the P-value threshold; it is equal to one minus the positive false discovery rate [21].

This gives

$$P(A|p < T) = \frac{\text{power} \times P(A)}{\text{power} \times P(A) + T \times (1 - P(A))} \qquad \text{(S18)}$$

Equation (3) of **Methods** is obtained by rewriting (S18) on the odds scale.

Formula (S18) can be interpreted either in a Bayesian setting, in which $P(A)$ reflects the prior belief in association between v and w, or in a frequentist framework in which the variants v and w are assumed to be sampled from a set of variants. $P(A)$ then reflects the frequency of true associations in this set.

Calculating the power term in (S18) requires making assumptions about the variant frequencies and the true association effect size. We illustrate this with a simple approximate calculation as follows. Suppose v and w respectively have frequencies f and g and suppose the total sample size is *N*. We use a previously derived expression[22] for the variance of the estimated log-odds ratio assuming an additive effect:

$$V \approx \frac{1}{2Nf(1-f)g(1-g)} \qquad \text{(S19)}$$

We note that the accuracy of (S19) is expected to be greatest for variants at intermediate frequencies and for small effect sizes[22] but we adopt this here for illustration purposes. As in (S12)-(S14) under asymptotic assumptions the effect size estimate θ will distributed around the true effect size θ_0 with this variance

$$\theta \sim N(\theta_0, V) \qquad \text{(S20)}$$

The P-value can then be computed by formula (S14). **Supplementary Figure 4** depicts the resulting association test power (dashed lines) and the posterior probability of association (left-hand side of (S18), solid lines) for two choices of association effect size distribution (odds ratio = 2, or odds ratio = 4) under a range of variant frequencies and prior probabilities.

2.5.2. Interpretation of Bayes factors

Since our study data has already been observed, it is appropriate to conduct a calculation similar to (S18) conditioned on the observed study data. This leads to:

$$P(A|\text{data}) = \frac{P(\text{data}|A) \cdot P(A)}{P(\text{data}|A) \cdot P(A) + P(\text{data}|!A) \cdot P(!A)}$$

Since the Bayes factor is $P(\text{data}|A)/P(\text{data}|!A)$, this equation can more simply be expressed in terms of the Bayes factor as

$$P(A|\text{data}) = \frac{BF \cdot P(A)}{BF \cdot P(A) + (1 - P(A))} \quad \text{(S21)}$$

or on the odds scale as

$$\text{posterior odds}(A|\text{data}) = BF \times \text{prior odds}(A) \quad \text{(S22)}$$

As detailed above, we have used a log F(2,2) distribution to model effect sizes in our Bayes factor computation; this assumes that most association effect sizes concentrate near zero but also allows for relatively large effects with nontrivial probability. Under this assumption (S21-S22) can be used to directly interpret Bayes factors in terms of evidence for association; a possible calculation is set out in **Methods**.

3. Supplementary Text

3.1. Investigation of additional signals of association

3.1.1. Overall interpretation of additional signals

In addition to the HbS associations described in main text, we observed additional candidate associations between other human and *Pf* variants in our discovery data. The evidence for these associations is likely not strong enough to establish these associations without additional information. A full list of associations can be found in **Supplementary Table 1**; we detail those with $BF > 10^5$ and those involving other previously established human protective mutations at the *ABO*, *ATP2B4* and glycophorin regions below. Additionally, we also observed a larger number of *Pf* variants associated at lower levels of evidence ($BF > 10^3$) with HbS and we interpret these below.

3.1.2. Association between *GCNT2* and two regions of the *Pf* genome

We observed a candidate host-parasite genetic association intronic variation in the gene *GCNT2* (lead SNP: rs517371 chr6:10,554,048 C>G) and a non-synonymous SNP in MSP4 (*Pf* chr2:278,302 T>C). (BF=2.8x10^6; P = 1.4x10^{-9}; OR = 0.39 (0.28-0.53) for effect of human 'G' allele on parasite 'C' allele; **Supplementary Table 1**). *GCNT2* determines the two reciprocal antigens of the I blood group by adding a β1,6-linked polylactosamine side chain onto the i antigen to generate a branched I antigen. Besides newborns and individuals with rare inactivating mutations in *GCNT2*, all individuals express both I and i antigens on the erythrocyte surface to varying degrees, with I expression dominating [23]. *GCNT2* has three alternative first exons that have cell-type specific expression. The associated variants lie just upstream of the 2nd first exon that is expressed in multiple cell types, but not in erythrocytes which are thought to use the 3rd first exon isoform [24]. It is thus unclear whether these variants affect gene or transcript expression in relevant cell types. P. falciparum's MSP proteins are generally thought to act in early stages of RBC invasion, but the specific function of MSP4 remains unknown [7].

Interestingly, an additional signal of association between *GCNT2* variation (rs78972384 C > T) and *Pf* variation in *RH1* (chr:138,623 A > T) was also observed (BF=4.9; 1.5x10^{-6}). We are unaware of any existing evidence for a molecular interaction between the I antigen with *PfMSP4* or *PfRH1*, or with parasite invasion more generally.

3.1.3. Association between HLA alleles and variation in several regions of the *Pf* genome

As shown in **Supplementary Table 1**, a number of HLA alleles associate with variation in the parasite genome with Bayes factors in the range $10^5 – 10^6$. These include including HLA-A*68 (associated with variation in *PfWDTC1* "WD and tetratricopeptide repeats protein 1, putative"); HLA-B*49 (associated with variation in *PF3D7_1141700* "OTU domain-containing protein, putative"); HLA-DQB1*03 (associated with variation in *PF3D7_0714900* "tRNA Serine"); HLA-A*01 (associated with variation in *PfXL2* "Exported lipase 2"); and HLA-DPA*02 (associated with variation in *PfSET3* "SET domain protein, putative"). We caution that HLA alleles in our data were obtained by imputation using a reference panels with limited coverage of African populations[9]; however, the alleles listed here have reasonably allele frequency (> 4% in both countries) and reasonable imputation confidence (IMPUTE info > 0.94 in both countries).

Among these candidate associations, the association between HLA-A*01 and *Pf* chr10:86,025 A>T may be notable because the *Pf* 'A' allele was only observed in infections of individuals that do not carry the HLA-A*01 allele, leading to a large estimated effect size (OR= 0.06).

3.1.4. Association with ABO, ATP2B4 and glycophorin variation

We note here weak evidence in our discovery data (BF > 10^3) for association between previously established host protective mutations other than HbS[9], and *Pf* variation. We observed weak evidence for association of the O blood group mutation (rs8176719) and an intergenic variant near *PfAROM* (Pf chr2:257614 C>G; BF = 1.6×10^4). The *ATP2B4* variant rs4951377 was associated with an intergenic variant on *Pf* chromosome 7 (chr7: 507,739 G > A; BF = 7.3×10^3). The glycophorin structural variant DUP4 was not associated with any *Pf* variants with BF > 10^3.

3.1.5. Additional associations with HbS

In addition to the *Pfsa1*, *Pfsa2* and *Pfsa3* loci described in main text, a number of other *Pf* loci appear to be associated with HbS at lower levels of evidence (*BF* > 10^3; **Supplementary Table 1**). These include missense variants in both *PfCLAG3.1* and *PfCLAG3.2* (cytoadherence-linked asexual protein 3) on chromosome 3, in *PfLSA1* (Liver-stage antigen 1), a noncoding variant close to *PfREX2* (Ring-exported protein 2) and variation in *PfEBL1* (erythrocyte binding like protein 1). These variants are also among those observed to be in strong LD with *Pfsa* variants (**Supplementary Table 3**) and we interpret them as likely reflecting the same association signal.

3.1.6. Investigation of HbS-MSP1 association

A previous study [25] reported evidence of association between HbS and specific alleles of *P.falciparum* MSP1 ("merozoite surface protein 1", *PF3D7_0930300*) in asymptomatic infections from Dielmo, Senegal (N=77) and Dienga, Gabon (N=163). In both cohorts, the study found decreased frequency of MAD20 alleles in HbAS individuals compared to HbAA individuals. Specific MSP1 alleles, including K1-d and K1-e were also observed exclusively either in HbAA or HbAS individuals in the smaller Senegal cohort, but these alleles were not observed in the Gabon cohort.

In our study we found no strong evidence of association between HbS and MSP1 variant (maximum BF_{HbS} = 94 for variants in *PF3D7_0930300* or 2kb flanking region), and thus our data do not appear to replicate the above associations. We note several possible reasons for this. First, the original study noted several complex features, including high occurrence of infections with multiple MSP1 genotypes (based on multiple PCR bands), and additional epidemiological features associated with MSP1 types (including with MAD20 alleles) in the Senegal cohort. The sample size was also relatively limited and a number of comparisons were carried out. It is thus possible that the findings are specific to the cohorts studied. However, the MSP1 locus is highly polymorphic and carries multiple allelic forms that were assessed via PCR approaches in the above study; moreover these display a form of dipmorphism in the sense that they have seldom or rarely been observed to recombine [26]. Our results, which are based on mapping of sequence reads to the Pf3D7 reference genome (which carries the MAD20 allele) may not capture the relevant genetic variation. In general, specialised methods (such as those based on a population reference graph [27]) will likely be needed to fully resolve variation in highly polymorphic or dimorphic regions.

3.2. Functional information on the *Pfsa* loci

3.2.1. Relevant gene and protein identifiers

The *Pfsa* loci described in main text include non-synonymous mutations of three *P.falciparum* genes: *PF3D7_0215300* (*PfACS8* "acyl-CoA synthetase"), *PF3D7_0220300* ("Plasmodium exported protein, unknown function") and *PF3D7_1127000* ("protein phosphatase, putative") (**Figure 1**). The following table details the name and relevant identifier symbols for these genes.

Locus	Gene identifier and name	Alternate identifiers	Protein identifier
Pfsa1	*PF3D7_0215300* (ACS8; "Acyl co-A synthetase")	*PF02_0144*; *PFB0695c*	O96232
Pfsa2	*PF3D7_0220300* ("Plasmodium exported protein, unknown function")	*PFB0923c*	Q8I654

Pfsa3	*PF3D7_1127000* ("Protein phosphatase, putative")	*PF11_0281*	Q8II93

In addition to these genes, we note in main text that available genome assemblies from *Pfsa3+* parasites contain a structural rearrangement of the Pfsa3 region which appears to duplicate part of the gene *SNRPF* (**Extended Data Figure 9** and **Supplementary Figure 3**).

3.2.2. Information on protein function

We detail known functional information relating to the above genes.

PF3D7_0215300*:* *PfACS8* is a member of the Acyl co-A synthetase family, which includes 13 genes in *P.falciparum* but varies in number of paralogs between other laverania[28,29]. *ACS8* and a number of other ACS genes are thought to have arisen as duplicates of *ACS9* [28]. Acyl-coA synthetases play roles in fatty acid metabolism and particular in activation of fatty acids scavenged from the host cytosol[30]. They are expressed in intraerythrocytic blood stages as detailed further below. ACS8 and ACS9 in particular have been predicted to localise to the apicoplast membrane based on the presence of a bipartite leader sequence [31], but to our knowledge their cellular location and function has not been directly observed [32]. This is distinct from other ACS proteins which are thought to localise to the erythrocyte cytoplasm [33]. Previous doctoral work used CRISPR-Cas9 to generate an in vitro knockout for *ACS8* and other ACS genes from the 3D7 lab strain, and used this to study growth phenotypes under conditions of glucose depletion and enriched for specific fatty acids [34]; this suggested ACS8 is nonessential for intraerythrocytic growth, as only modest variation in growth rates was observed, with some decrease in growth rates of the ACS8 knockout observed in low-glucose conditions. Consistent with this, ACS8 was not found to be essential for in vitro growth in a recent screen using randomly inserted *piggyBac* transposons to knockdown genes [35] genome-wide, detailed further below.

PF3D7_0220300*:* Q8I654 (encoded by *PF3D7_0220300*) was previously computationally identified as an exported protein (meaning that it is exported from the parasite to the host cytosol during blood-stage infections) due to the presence of a PEXEL motif in its amino acid sequence and an appropriate signal peptide sequence [36,37]. We describe the PEXEL motif and its sequence context further below. Export of Q8I654 to the erythrocyte cytosol was confirmed in doctoral work by Tamira K. Butler described previously[38]. In that work, cultured 3D7 parasites, and 3D7 parasites modified to conditionally express a recombinant version of Q8I654 with a fluorescent tag incorporated at the C-terminal end, were used to investigate the expression and localisation of Q8I654 in in vitro blood stage infections. Q8I654 was observed to localise to vesicles that appeared distinct from either Maurer's clefts or J-dots. It was further reported that Q8I654 acts to sequester the host protein stomatin within these vesicles.

Butler also found that Q8I654 growth differed in conditional knockout lines depending on conditions[38] and therefore suggested that Q8I654 is essential to in vitro growth of parasites, through a putative role in glucose uptake. However, this result contrasts with findings from the *piggyBac* screen [35] described further below, and other knockdown experiments that have been performed (Daniel Goldberg, personal communication). These experiments suggest that *PF3D7_0220300* is likely not to be essential to in vitro growth and may be unrelated to glucose uptake, except perhaps under particular conditions.

PF3D7_112700*:* Q8II93 encoded by *PF3D7_112700* has been identified as a putative protein phosphatase due to the presence of a PTP Tyrosine Phosphatase-like group in its amino acid sequence [39]. However, it was reported as low-scoring in that analysis and it was further noted that its PTP domain clustered separately to PTP domains from other Eukaryotes, suggesting it is somewhat unusual. Q8II93 has also been identified as an exported protein[36] and contains a non-canonical PEXEL motif which we detail further below. Using mass spectrometry, Q8II93 was also identified as one of 116 proteins present in the *P.falciparum* food vacuole [40].

3.2.3. Dispensability of HbS-associated genes

A recent study used *piggyBac* transposons to randomly introduce mutations to 3D7 genome-wide, and analysed viability for *in vitro* growth of the mutant parasites [35]. The table below details the results of this study for the three genes of interest. Notably, the study found viable parasites containing insertions upstream of the PEXEL motif in both *PF3D7_0220300* and *PF3D7_1127000*, suggesting

these genes (and their encoded proteins) are not essential for blood stage growth of 3D7-derived parasites.

Locus	Gene identifier, strand, and transcription and CDS locations	# of *piggyBac* sites	Observed recognition sites	Mutagenesis index / mutant growth fitness scores; study conclusion
Pfsa1	*PF3D7_0215300* (chr2; -ve strand) 628,091 - 632,681 [628,639 – 631,305]	38	**629083**; **631100**; *631886*; *632052*; *632086*; *632350*; *632390*; *632621*; *632662*	1 / -1.796; Mutable in coding sequence
Pfsa2	*PF3D7_0220300* (chr2; -ve strand) 812,892 – 815,853 [813,845 – 814,672]	10	*813018*; *813355*; *813693*; **814310***; *815013*; *815610*; *815757*	0.817 / -2.119 Mutable in coding sequence
Pfsa3	*PF3D7_1127000* (chr11; -ve strand) 1,055,701 – 1,058,777 [1,056,199 – 1,058,055]	20	*1055840*; **1057481**†; **1057630**†; 1057814; 1058384; *1058543*	1 / -0.05 Mutable in coding sequence

Table: detail of previously reported dispensability data[35] for Pfsa genes based on a *piggyBac* screen. Columns show the region and gene identifier and chromosome of the relevant gene; location of gene on chromosome including annotated transcribed sequence and coding sequence (CDS; in square brackets) on the 3D7 reference genome; number of piggyBac recognition (*TTAA*) sites in coding sequence; the location of piggyBac recognition sites observed in the screen[35], with sites in the untranslated sequence denoted in italics and sites in coding sequence in bold. Asterisk denotes a site inside a PEXEL motif and dagger denotes site upstream of a PEXEL motif. The last column reports the mutagenesis and mutant growth scores and the conclusion reported by the study[35].

3.2.4. Relationship of *Pfsa1*+ and *Pfsa2*+ alleles to PEXEL motifs

As described above, both *PF3D7_0220300* and *PF3D7_1127000* are identified as exported proteins based on the presence of PEXEL motifs within their amino acid sequence. We review background on PEXEL-mediated protein export in this section, and describe the relationship of the HbS-associated alleles in the *Pfsa2* and *Pfsa3* regions to the motifs. The PEXEL motif (reviewed recently[36]) is a sequence of amino acids of the form RxLxE/Q/D (in which the capital letters follow the IUPAC code and x stands for an arbitrary amino acid [41,42]) which is known to mediate export of the protein from the parasite to the erythrocyte cytosol. Slightly altered 'non-canonical' PEXEL motifs also mediate export in some proteins [36,43]. Export of PEXEL-containing proteins is a multi-step process, with a first step involving initial cleavage of the motif by plasmepsin V (PMV) in the parasite endoplasmic reticulum [44]. This cleavage leaves the last two residues of the motif (i.e. xE/Q/D) at the N-terminus of the cleaved protein.

Experimental studies using engineered recombinants of known exported proteins (including *PfEMP1* and *KAHRP*) have indicated that while this cleavage by PMV is necessary for export, it is not sufficient. Several additional factors also appear to determine successful export, including the position of the motif relative to signal peptide within the protein[45] and the sequence of amino acids immediately downstream (C-terminal) of the PEXEL[46,47], although to our knowledge the precise conditions for protein export have not been discovered. It has also been reported that export of mature PEXEL proteins (i.e. proteins following cleavage by PMV), and of exported proteins that lack a PEXEL motif (PNEPs), use a common pathway that depends substantially on the remaining N-terminal sequence [47]. It is therefore possible that export is affected by genetic variation within or immediately downstream of the motif.

The following table indicates the position of the HbS-associated SNPs (Supplementary Table 1) in *PF3D7_0220300* and *PF3D7_112700* in relation to the PEXEL motif in these genes. It is notable that the HbS-associated variants lie close to the PEXEL motif and affect bases immediately downstream (in an immediately adjacent amino acid for *PF3D7_1127000*, and amino acids both up- and downstream of the PEXEL for *PF3D7_0220300*). This therefore raises the question of whether the *Pfsa*+ alleles might alter export of these proteins.

Locus	Gene identifier, base pair and amino acid location of PEXEL motif in Pf3D7	MOTIF sequence	*Pfsa* mutation	Codon change
Pfsa2	*PF3D7_0220300*<br>Pf3D7_02_v3<br>814,307 - 814,321<br>AA: 49-53	RTLTE	314,288 C>T<br>(+7)	GAT -> AAT<br>(Aspartic acid -> Asparagine)
			314,329 A>G<br>(-3)	ATA -> ACA<br>(Isoleucine -> Threonine)
Pfsa3	*PF3D7_1127000*<br>Pf3D7_11_v3<br>1,057,438 – 1,057,452<br>AA: 71-75	RCLNY*	1,057,437 T > C<br>(+1)	AAA -> GAA<br>(Lysine -> Glutamic acid)

Table: detail of HbS-associated SNPs (Supplementary Table 1) in protein coding sequence within 50bp of a PEXEL motif. Columns show the locus name; the gene identifier, motif sequence (asterisk denotes the sequence matches a non-canonical motif [36]), location of the nucleotide sequence of the motif within the 3D7 reference sequence, and location in terms of amino acids within the translated protein; the base pair location of the mutation (from Supplementary Table 1), with numbers in brackets indicating the amino acid location upstream (-ve numbers) or downstream (+ve numbers) of the PEXEL motif; and the corresponding amino acid change.

3.2.5. *Pfsa* gene expression in 3D7 parasites

Several datasets on mRNA expression in the 3D7 lines of *P.falciparum* have been generated. We examined data on asexual blood stage infections [48-51], and on gametocyte and mosquito-borne stages [52]. We also examined transcripts identified using long-read sequencing of mixed asexual blood stage parasites [53]. Using these datasets we noted the following features. Some expression of all three genes was detected in gametocytes and sporozoites[52]. Expression was also observed in multiple asexual blood stages [48,50,51]. Across asexual blood stages we noted that expression of *ACS8* / *PF3D7_0215300* was generally highest in early-stage parasites (5-20 hours following invasion; representing rings and early trophozoites; **Extended Data Figure 7**). Expression of *PF3D7_0220300* was relatively constant but with some evidence for increased expression at trophozoite stages (**Extended Data Figure 7**). In these experiments using 3D7, *PF3D7_1127000* was expressed at similar levels across all blood stage timepoints. The long-read sequencing dataset[53] also reports an alternate transcript of *PF3D7_1127000* in which transcription appears to continue through the first intron.

3.2.6. Increased expression of *PF3D7_1127000* in *Pfsa*+ parasites

In main text we describe a recent study which analysed RNA transcription levels of *Pf* genes in a sample of HbAA and HbAS children from Mali, all of whom were ascertained with uncomplicated malaria infections[49]. This study found that *PF3D7_1127000* has higher mRNA expression levels among trophozoite-stage infections of HbAS children than in the HbAA children ($\log_2$ fold change of expression in HbAS versus HbAA children = 5.0; $P = 1.08 \times 10^{-18}$; computed using DESeq2[49]). Our results raise the question of whether the expression change may be driven by a genetic change in parasites linked to the *Pfsa3+* allele.

To investigate this we re-examined the data from Saelens et al[49] as follows. First, we examined RNA-seq reads aligning to the three lead HbS-associated SNPs the *Pfsa1-3* loci and used these to call genotypes (detailed in **Supplementary Table 6**). The *Pfsa1+* and *Pfsa3+* alleles were strongly associated with HbS in these samples (OR = 15.2, 95% CI =2.7 – 87.0, P = 2.2×10^{-3} for *Pfsa1+*; OR = 58.9, 95% CI = 5.9 – 590.0, P = 5.31×10^{-4} for *Pfsa3+*) as detailed in **Supplementary Table 2**. The *Pfsa2+* allele was not observed in these samples; this presumably reflects the low population frequencies of this allele in Mali (**Figure 3**) but we also noted that the *Pfsa2* SNP was covered by relatively few reads in several samples (**Supplementary Table 6**), suggesting low expression levels and leading to missing genotype calls. These results therefore provide replication of the HbS-*Pfsa* association described in main text in this set of uncomplicated malaria samples.

Given that HbAS and *Pfsa3+* are associated in these samples, we considered whether the upregulation of *PF3D7_1127000* might be better explained by *Pfsa3+* genotypes than HbAS status in these samples We noted that the genotype at the second-most HbS-associated SNP in this region (chr11:1,057,437 T>C) was a particularly good predictor of *PF3D7_1127000* expression (**Extended Data Figure 6** and **Supplementary Table 6**). This improvement is due to two individuals for which the expression of *Pf3D7_1127000* appear inconsistent with a model where high expression is due to the HbAS genotype. First, an HbAS individual (AS08) who was reported as having trophozoite-stage parasites and *Pfsa3-* genotype at the chr11:1,057,437 SNP, showed the lowest expression of *PF3D7_1127000* among all HbAS samples in the data (transcripts per kilobase million (TPM) = 28, versus TPM > 140 for all other HbAS samples; TPM > 1390 for all other HbAS samples with trophozoite-stage infections). Secondly, a single HbAA individual (AA01) which had predominantly *Pfsa3+* genotype had the highest expression of *PF3D7_1127000* among all HbAA samples (TPM = 253, versus TPM < 155 for all other HbAA samples), despite being identified as a ring stage infection.

The data above has limited power to detect differences in the possible causes of upregulation. However, we also analysed a second experiment from the same study[49] in which both 3D7 and the Uganda Palo Alto isolate[54] (FUP/H) were studied in a time course for 48 hours post-invasion. This experiment is relevant because FUP/H carries all three *Pfsa+* alleles, including the C allele at chr11:1,057,437 T>C (inferred from available sequencing data as described in **Methods**.) In the experiment, culture was carried out in blood from both HbAA or HbAS individuals. In all blood samples, expression patterns of *PF3D7_1127000* differed between 3D7 and FUP/H parasites (**Extended Data Figure 7**): strong expression was observed for *PF3D7_1127000* in trophozoite-stage infections of FUP/H in all blood samples studied (e.g. TPM > 1,000 for FUP/H infections at 24-30 hours post-infection) while expression was relatively weak in 3D7 infections at the same time points (TPM < 60). **Extended Data Figure 8** further details this behaviour in comparison to other *Pf* genes in the two HbAA blood samples analysed. This experiment therefore also supports a model in which the *Pfsa+* alleles (presumably *Pfsa3+*) cause upregulation of *PF3D7_1127000*, as opposed to upregulation in response to the HbAS genotype directly.

We note that in the data from Mali, one HbAS sample (AS15) appears to carry opposing alleles (i.e. *Pfsa3-* and *Pfsa3+* respectively) at the chr11:1,058,35 and chr11:1,057,437 variants. This sample had high expression of *PF3D7_1127000*. If the *Pfsa3+* are genuinely the cause of increased expression as suggested above, this might suggest the chr11:1,057,437 SNP is the key functional mutation. However, the *Pfsa3+* mutations are also linked to a nearby structural variant (**Extended Data Figure 9** and **Supplementary Figure 3**) and further work will be needed to determine the functional elements.

Finally, for completeness, we note that this experiment also suggests that a similar differential expression effect may occur for *PF3D7_0220300* (**Extended Data Figure 7**). *PF3D7_0220300* in this experiment was expressed at substantially lower levels in FUP/H than in 3D7 during ring stages, but has equal or higher expression at trophozoite stages.

3.3. Linear mixed-model based analysis of HbS-*Pfsa* association

Motivated by a reviewer comment, we further explored the use of a linear mixed model (LMM) to assess the association between HbS and the *Pfsa* alleles. Specifically we used FaST-LMM [55] to assess association in the 3,346 discovery samples (as used in **Figure 1),** working separately in each population and including both a parasite genetic relatedness matrix (*Pf* GRM) and human genetic relatedness matrix (human GRM) to model random effects. To compute *Pf* GRMs, we used the same sets of SNPs as used for principal components computation (as detailed in **Methods** and shown in **Extended Data Figure 3**). To compute the human GRM, we used the set of directly-typed variants that passed QC in our previously reported genome-wide association study [9], after excluding variants with minor allele frequency < 1%, variants on the sex chromosomes or in the first 10Mb of chromosome 11 (which contains the *HBB* gene and HbS allele), and after thinning variants to no closer than 0.02cM apart using the HapMap combined recombination map. We ran FaST-LMM including only the human GRM, or both the human and *Pf* GRM, for each set of *Pf* variants considered. For comparison we also used the same data to fit basic linear and logistic regression models without any additional covariates in each country.

The table below reports the results of this analysis. In general, we found that results were qualitatively similar to those reported in **Extended Data Figure 3** (based on logistic regression with principal components). Specifically, inclusion of the human GRM had little effect on the association test

compared to an unadjusted linear model, but inclusion of a GRM computed from *Pf* genetic variants reduced the association signal to a limited extent. As in **Extended Data Figure 3,** exclusion of the *Pfsa* region variants from the *Pf* GRM partly restored this association. However, these results also highlight a key issue when applying the LMM approach to these data: we found that P-values from the LMM were systematically and substantially lower than those from logistic regression. (For example, P < $6x10^{-18}$ at *Pfsa1* for all versions of the linear mixed model test in Kenya, compared to P = $2x10^{-11}$ using unadjusted logistic regression). Similarly low P-values were observed when using a linear model without random effects. Our interpretation is that the P-values from the linear models strongly overstate the true statistical evidence, and that this arises because of the model misspecification inherent in applying the homoscedastic Gaussian error model to binary outcome data in the presence of large effects.

Country	*Pf* GRM variants	Human GRM variants	P-value	Effect size	Std. error	Heritability	*Pf* / hs mixing
Pfsa1 (chr2:631,190 T > A)							
Gambia	All variants	All	1.48E-03	0.0192	0.0060	1.00	1.00
	Exc. *Pfsa* regions	All	3.14E-04	0.0249	0.0069	1.00	1.00
	Exc. chr 2 and 11	All	3.30E-04	0.0257	0.0071	1.00	1.00
	No *Pf* GRM	All	7.89E-06	0.0470	0.0105	0.09	0.00
(*)	None (normal linear model)		8.20E-06	0.0464	0.0104		
(**)	None (logistic regression model)		2.58E-04	0.2145	0.0587		
Kenya	All variants	All	5.90E-18	0.0687	0.0078	1.00	1.00
	Exc. *Pfsa* regions	All	2.50E-21	0.0826	0.0085	1.00	1.00
	Exc. chr 2 and 11	All	5.13E-22	0.0856	0.0087	1.00	0.88
	No *Pf* GRM	All	3.09E-25	0.0990	0.0093	0.72	0.00
(*)	None (normal linear model)		3.18E-26	0.1006	0.0093		
(**)	None (logistic regression model)		2.27E-11	0.5687	0.0850		
Pfsa2 (chr2:814,288 C > T)							
Gambia	All variants	All	6.46E-01	-0.0006	0.0014	1.00	1.00
	Exc. *Pfsa* regions	All	6.56E-01	-0.0006	0.0015	1.00	1.00
	Exc. chr 2 and 11	All	7.53E-01	-0.0005	0.0015	1.00	1.00
	No *Pf* GRM	All	6.63E-01	-0.0010	0.0023	0.34	0.00
(*)	None (normal linear model)		6.73E-01	-0.0010	0.0024		
(**)	None (logistic regression model)		9.89E-01	-1.3299	94.39		
Kenya	All variants	All	2.83E-11	0.0609	0.0091	1.00	1.00
	Exc. *Pfsa* regions	All	1.31E-14	0.0777	0.0099	1.00	1.00
	Exc. chr 2 and 11	All	4.10E-15	0.0796	0.0100	1.00	1.00
	No *Pf* GRM	All	1.20E-16	0.0915	0.0109	0.34	0.00
(*)	None (normal linear model)		4.46E-17	0.0923	0.0108		
(**)	None (logistic regression model)		2.63E-09	0.4778	0.0803		
Pfsa3 (chr11:1,058,035 T > A)							
Gambia	All variants	All	4.54E-02	0.0138	0.0069	1.00	1.00
	Exc. *Pfsa* regions	All	1.96E-02	0.0176	0.0075	1.00	1.00
	Exc. chr 2 and 11	All	9.21E-03	0.0207	0.0079	1.00	1.00
	No *Pf* GRM	All	2.06E-04	0.0437	0.0118	0.02	0.00
(*)	None (normal linear model)		2.07E-04	0.0425	0.0114		
(**)	None (logistic regression model)		2.77E-03	0.2313	0.0773		
Kenya	All variants	All	5.84E-18	0.0727	0.0083	1.00	1.00
	Exc. *Pfsa* regions	All	2.38E-22	0.0886	0.0089	1.00	1.00
	Exc. chr 2 and 11	All	1.72E-22	0.0892	0.0090	1.00	1.00
	No *Pf* GRM	All	3.83E-24	0.1017	0.0098	0.34	0.00
(*)	None (normal linear model)		6.25E-25	0.1075	0.0102		
(**)	None (logistic regression model)		1.55E-11	0.5995	0.0889		

Table: comparison of linear mixed model, linear and logistic regression analysis of HbS-*Pfsa* association. Columns show: the *Pfsa* region name and variant tested, the population, an indicator of the variants included in the *Pf* GRM and the human GRM for linear mixed model (LMM) analysis, the P-value, estimated effect size and standard error; the estimated 'heritability' parameter indicating the contribution of both GRMs to the model fit (on a scale of 0-1) for LMM analysis; and the estimated mixing parameter determining the relative contribution of the parasite versus the human GRM to the model fit (on a scale of 0-1) for LMM analysis. All LMM analysis was conducted using FaST-LMM. Rows marked (*) are estimated using a linear regression model in R with no additional covariates. Rows marked (**) are

estimated using logistic regression in R with no additional covariates. For comparison with FaST-LMM, predictor (HbS) genotypes were standardised to have mean 0 and empirical variance 1 prior to model fit.

We note that the magnitude of the HbS-*Pfsa* association described here may be relatively extreme, and that linear mixed models have elsewhere been shown to work well for binary outcomes with balanced outcome frequencies and relatively small genetic effect sizes [56]. However, the strength of association between host and pathogen genotypes is at present unknown for most infectious diseases, and the frequency of the potentially relevant pathogen variants also varies widely. These results suggest that some caution may be needed in interpreting linear mixed model results in such settings.

4. Supplementary references

1 Otto, T. D. *et al.* Long read assemblies of geographically dispersed Plasmodium falciparum isolates reveal highly structured subtelomeres. *Wellcome Open Res* **3**, 52, doi:10.12688/wellcomeopenres.14571.1 (2018).

2 Ndila, C. M. *et al.* Human candidate gene polymorphisms and risk of severe malaria in children in Kilifi, Kenya: a case-control association study. *Lancet Haematol* **5**, e333-e345, doi:10.1016/S2352-3026(18)30107-8 (2018).

3 Bohme, U., Otto, T. D., Sanders, M., Newbold, C. I. & Berriman, M. Progression of the canonical reference malaria parasite genome from 2002-2019. *Wellcome Open Res* **4**, 58, doi:10.12688/wellcomeopenres.15194.2 (2019).

4 Oyola, S. O. *et al.* Whole genome sequencing of Plasmodium falciparum from dried blood spots using selective whole genome amplification. *Malaria Journal* **15**, 597, doi:10.1186/s12936-016-1641-7 (2016).

5 Ahouidi, A. *et al.* An open dataset of Plasmodium falciparum genome variation in 7,000 worldwide samples [version 1; peer review: awaiting peer review]. *Wellcome Open Research* **6**, doi:10.12688/wellcomeopenres.16168.1 (2021).

6 Miles, A. *et al.* Indels, structural variation, and recombination drive genomic diversity in Plasmodium falciparum. *Genome Res* **26**, 1288-1299, doi:10.1101/gr.203711.115 (2016).

7 Cowman, A. F., Tonkin, C. J., Tham, W. H. & Duraisingh, M. T. The Molecular Basis of Erythrocyte Invasion by Malaria Parasites. *Cell Host Microbe* **22**, 232-245, doi:10.1016/j.chom.2017.07.003 (2017).

8 Binks, R. H. *et al.* Population genetic analysis of the Plasmodium falciparum erythrocyte binding antigen-175 (eba-175) gene. *Mol Biochem Parasitol* **114**, 63-70, doi:10.1016/s0166-6851(01)00240-7 (2001).

9 Band, G. *et al.* Insights into malaria susceptibility using genome-wide data on 17,000 individuals from Africa, Asia and Oceania. *Nature Communications* **10**, 5732, doi:10.1038/s41467-019-13480-z (2019).

10 Hamilton, W. L. *et al.* Extreme mutation bias and high AT content in Plasmodium falciparum. *Nucleic Acids Res* **45**, 1889-1901, doi:10.1093/nar/gkw1259 (2017).

11 Pearl, J. *Causality : models, reasoning, and inference*. (Cambridge University Press, 2000).

12 Berkson, J. Limitations of the application of fourfold table analysis to hospital data. *Int J Epidemiol* **43**, 511-515, doi:10.1093/ije/dyu022 (2014).

13 Timmann, C. *et al.* Genome-wide association study indicates two novel resistance loci for severe malaria. *Nature* **489**, 443-446, doi:10.1038/nature11334 (2012).

14 Malaria Genomic Epidemiology Network. Reappraisal of known malaria resistance loci in a large multicenter study. *Nat Genet* **46**, 1197-1204, doi:10.1038/ng.3107 (2014).

15 Band, G. *et al.* A novel locus of resistance to severe malaria in a region of ancient balancing selection. *Nature* **526**, 253-257, doi:10.1038/nature15390 (2015).

16 Leffler, E. M. *et al.* Resistance to malaria through structural variation of red blood cell invasion receptors. *Science* **356**, doi:10.1126/science.aam6393 (2017).

17 Clayton, D. Link functions in multi-locus genetic models: implications for testing, prediction, and interpretation. *Genet Epidemiol* **36**, 409-418, doi:10.1002/gepi.21635 (2012).
18 Greenland, S. & Mansournia, M. A. Penalization, bias reduction, and default priors in logistic and related categorical and survival regressions. *Statistics in Medicine* **34**, 3133-3143, doi:10.1002/sim.6537 (2015).
19 Wakefield, J. Bayes factors for genome-wide association studies: comparison with P-values. *Genet Epidemiol* **33**, 79-86, doi:10.1002/gepi.20359 (2009).
20 Nocedal, J. & Wright, S. J. *Numerical optimization*. 2nd edn, (Springer, 2006).
21 Storey, J. D. The positive false discovery rate: a Bayesian interpretation and the <i>q</i>-value. *The Annals of Statistics* **31**, 2013-2035, doi:10.1214/aos/1074290335 (2003).
22 Vukcevic, D., Hechter, E., Spencer, C. & Donnelly, P. Disease model distortion in association studies. *Genetic epidemiology* **35**, 278-290, doi:10.1002/gepi.20576 (2011).
23 Reid, M. E. The gene encoding the I blood group antigen: review of an I for an eye. *Immunohematology* **20**, 249-252 (2004).
24 Cooling, L. An update on the I blood group system. *Immunohematology* **35**, 85-90 (2019).
25 Ntoumi, F. *et al.* Imbalanced distribution of Plasmodium falciparum MSP-1 genotypes related to sickle-cell trait. *Mol Med* **3**, 581-592 (1997).
26 Rich, S. M. & Ayala, F. J. Population structure and recent evolution of <em>Plasmodium falciparum</em&gt. *Proceedings of the National Academy of Sciences* **97**, 6994, doi:10.1073/pnas.97.13.6994 (2000).
27 Letcher, B., Hunt, M. & Iqbal, Z. Gramtools enables multiscale variation analysis with genome graphs. *Genome Biology* **22**, 259, doi:10.1186/s13059-021-02474-0 (2021).
28 Bethke, L. L. *et al.* Duplication, gene conversion, and genetic diversity in the species-specific acyl-CoA synthetase gene family of Plasmodium falciparum. *Mol Biochem Parasitol* **150**, 10-24, doi:10.1016/j.molbiopara.2006.06.004 (2006).
29 Otto, T. D. *et al.* Genomes of all known members of a Plasmodium subgenus reveal paths to virulent human malaria. *Nature Microbiology* **3**, 687-697, doi:10.1038/s41564-018-0162-2 (2018).
30 Matesanz, F., Téllez, M. a.-d.-M. & Alcina, A. The Plasmodium falciparum fatty acyl-CoA synthetase family (PfACS) and differential stage-specific expression in infected erythrocytes. *Molecular and Biochemical Parasitology* **126**, 109-112, doi:https://doi.org/10.1016/S0166-6851(02)00242-6 (2003).
31 Ralph, S. A. *et al.* Metabolic maps and functions of the Plasmodium falciparum apicoplast. *Nature Reviews Microbiology* **2**, 203-216, doi:10.1038/nrmicro843 (2004).
32 Shears, M. J., Botté, C. Y. & McFadden, G. I. Fatty acid metabolism in the Plasmodium apicoplast: Drugs, doubts and knockouts. *Molecular and Biochemical Parasitology* **199**, 34-50, doi:https://doi.org/10.1016/j.molbiopara.2015.03.004 (2015).
33 Matesanz, F., Durán-Chica, I. & Alcina, A. The cloning and expression of Pfacs1, a Plasmodium falciparum fatty acyl coenzyme A synthetase-1 targeted to the host erythrocyte cytoplasm11Edited by M. Yaniv. *Journal of Molecular Biology* **291**, 59-70, doi:https://doi.org/10.1006/jmbi.1999.2964 (1999).

34 Demas, A. R. Selection at Work in Plasmodium Falciparum: Lessons From the Expanded Acyl CoA Synthetase Gene Family and in Vitro Artemisinin Resistance. *PhD Thesis* (2016).
35 Zhang, M. *et al.* Uncovering the essential genes of the human malaria parasite Plasmodium falciparum by saturation mutagenesis. *Science* **360**, eaap7847, doi:10.1126/science.aap7847 (2018).
36 Jonsdottir, T. K., Gabriela, M., Crabb, B. S., F. de Koning-Ward, T. & Gilson, P. R. Defining the Essential Exportome of the Malaria Parasite. *Trends in Parasitology* **37**, 664-675, doi:https://doi.org/10.1016/j.pt.2021.04.009 (2021).
37 Sargeant, T. J. *et al.* Lineage-specific expansion of proteins exported to erythrocytes in malaria parasites. *Genome Biology* **7**, R12, doi:10.1186/gb-2006-7-2-r12 (2006).
38 Butler, T. K. An Exported Malaria Protein Regulates Glucose Uptake During Intraerythrocytic Infection. *Washington University in St. Louis* **PhD Thesis** (2014).
39 Wilkes, J. M. & Doerig, C. The protein-phosphatome of the human malaria parasite Plasmodium falciparum. *BMC Genomics* **9**, 412, doi:10.1186/1471-2164-9-412 (2008).
40 Lamarque, M. *et al.* Food vacuole proteome of the malarial parasite Plasmodium falciparum. *PROTEOMICS – Clinical Applications* **2**, 1361-1374, doi:https://doi.org/10.1002/prca.200700112 (2008).
41 Hiller, N. L. *et al.* A host-targeting signal in virulence proteins reveals a secretome in malarial infection. *Science* **306**, 1934-1937, doi:10.1126/science.1102737 (2004).
42 Marti, M., Good, R. T., Rug, M., Knuepfer, E. & Cowman, A. F. Targeting malaria virulence and remodeling proteins to the host erythrocyte. *Science* **306**, 1930-1933, doi:10.1126/science.1102452 (2004).
43 Schulze, J. *et al.* The Plasmodium falciparum exportome contains non-canonical PEXEL/HT proteins. *Molecular Microbiology* **97**, 301-314, doi:https://doi.org/10.1111/mmi.13024 (2015).
44 Russo, I. *et al.* Plasmepsin V licenses Plasmodium proteins for export into the host erythrocyte. *Nature* **463**, 632-636, doi:10.1038/nature08726 (2010).
45 Boddey, J. A. *et al.* Export of malaria proteins requires co-translational processing of the PEXEL motif independent of phosphatidylinositol-3-phosphate binding. *Nature Communications* **7**, 10470, doi:10.1038/ncomms10470 (2016).
46 Boddey, J. A. *et al.* Role of Plasmepsin V in Export of Diverse Protein Families from the Plasmodium falciparum Exportome. *Traffic* **14**, 532-550, doi:https://doi.org/10.1111/tra.12053 (2013).
47 Grüring, C. *et al.* Uncovering Common Principles in Protein Export of Malaria Parasites. *Cell Host & Microbe* **12**, 717-729, doi:https://doi.org/10.1016/j.chom.2012.09.010 (2012).
48 Otto, T. D. *et al.* New insights into the blood-stage transcriptome of Plasmodium falciparum using RNA-Seq. *Molecular Microbiology* **76**, 12-24, doi:https://doi.org/10.1111/j.1365-2958.2009.07026.x (2010).
49 Saelens, J. W. *et al.* Impact of sickle cell trait hemoglobin on the intraerythrocytic transcriptional program of Plasmodium falciparum. *mSphere* **6**, doi:10.1128/mSphere.00755-21 (2021).
50 Toenhake, C. G. *et al.* Chromatin Accessibility-Based Characterization of the Gene Regulatory Network Underlying Plasmodium falciparum Blood-Stage

Development. *Cell Host Microbe* **23**, 557-569 e559, doi:10.1016/j.chom.2018.03.007 (2018).

51 Wichers, J. S. *et al.* Dissecting the Gene Expression, Localization, Membrane Topology, and Function of the Plasmodium falciparum STEVOR Protein Family. *mBio* **10**, e01500-01519, doi:10.1128/mBio.01500-19 (2019).

52 Gómez-Díaz, E. *et al.* Epigenetic regulation of Plasmodium falciparum clonally variant gene expression during development in Anopheles gambiae. *Scientific Reports* **7**, 40655, doi:10.1038/srep40655 (2017).

53 Lee, V. V. *et al.* Direct Nanopore Sequencing of mRNA Reveals Landscape of Transcript Isoforms in Apicomplexan Parasites. *mSystems* **6**, e01081-01020, doi:10.1128/mSystems.01081-20 (2021).

54 Geiman, Q. M. & Meagher, M. J. Susceptibility of a New World Monkey to Plasmodium falciparum from Man. *Nature* **215**, 437-439, doi:10.1038/215437a0 (1967).

55 Lippert, C. *et al.* FaST linear mixed models for genome-wide association studies. *Nature Methods* **8**, 833-835, doi:10.1038/nmeth.1681 (2011).

56 Matti, P., Peter, D. & Chris, C. A. S. Efficient computation with a linear mixed model on large-scale data sets with applications to genetic studies. *The Annals of Applied Statistics* **7**, 369-390, doi:10.1214/12-AOAS586 (2013).
