# Supplementary material for: Malaria protection due to sickle haemoglobin depends on parasite genotype
Source: Nature. 2021 Dec 9;602(7895):106–11. doi: 10.1038/s41586-021-04288-3 (PMC8810385; doi:10.1038/s41586-021-04288-3)
Supplement: Supplementary file 2 — Reporting Summary [file 41586_2021_4288_MOESM2_ESM.pdf]

nature research

Corresponding author(s): Gavin Band
Kirk A. Rockett
Dominic P. Kwiatkowski

Last updated by author(s): Nov 5, 2021

Reporting Summary

Nature Research wishes to improve the reproducibility of the work that we publish. This form provides structure for consistency and transparency in reporting. For further information on Nature Research policies, see our Editorial Policies and the Editorial Policy Checklist.

Statistics

For all statistical analyses, confirm that the following items are present in the figure legend, table legend, main text, or Methods section.

n/a	Confirmed	
☐	☒	The exact sample size (*n*) for each experimental group/condition, given as a discrete number and unit of measurement
☒	☐	A statement on whether measurements were taken from distinct samples or whether the same sample was measured repeatedly
☐	☒	The statistical test(s) used AND whether they are one- or two-sided<br>*Only common tests should be described solely by name; describe more complex techniques in the Methods section.*
☐	☒	A description of all covariates tested
☐	☒	A description of any assumptions or corrections, such as tests of normality and adjustment for multiple comparisons
☐	☒	A full description of the statistical parameters including central tendency (e.g. means) or other basic estimates (e.g. regression coefficient) AND variation (e.g. standard deviation) or associated estimates of uncertainty (e.g. confidence intervals)
☐	☒	For null hypothesis testing, the test statistic (e.g. *F*, *t*, *r*) with confidence intervals, effect sizes, degrees of freedom and *P* value noted<br>*Give P values as exact values whenever suitable.*
☐	☒	For Bayesian analysis, information on the choice of priors and Markov chain Monte Carlo settings
☒	☐	For hierarchical and complex designs, identification of the appropriate level for tests and full reporting of outcomes
☐	☒	Estimates of effect sizes (e.g. Cohen's *d*, Pearson's *r*), indicating how they were calculated

Our web collection on statistics for biologists contains articles on many of the points above.

Software and code

Policy information about availability of computer code

Data collection	No software was used to collect the data used in this study.
Data analysis	The following software was used to analyse data in this study:<br>GATK v3.6.0 (http://gatk.broadinstitute.org)<br>PICARD v2.6.0 (https://broadinstitute.github.io/picard/)<br>SNAKEMAKE v5.11.1 (https://snakemake.readthedocs.io)<br>R version 3.4.3 (https://cran.r-project.org)<br>datasette version 0.39 (https://docs.datasette.io/en/stable/)<br>d3.js version 5.15.1 (https://d3js.org)<br>QCTOOL v2.0.8 (https://www.well.ox.ac.uk/~gav/qctool)<br>LDBIRD v2.1.9 (https://code.enkre.net/qctool)<br>HPTEST v2.1.9 (https://code.enkre.net/qctool)<br>STAR v2.7.3a (https://github.com/alexdobin/STAR)<br>Salmon v1.5.1 (https://combine-lab.github.io/salmon/)

For manuscripts utilizing custom algorithms or software that are central to the research but not yet described in published literature, software must be made available to editors and reviewers. We strongly encourage code deposition in a community repository (e.g. GitHub). See the Nature Research guidelines for submitting code & software for further information.

Data

Policy information about availability of data

All manuscripts must include a data availability statement. This statement should provide the following information, where applicable:

- Accession codes, unique identifiers, or web links for publicly available datasets
- A list of figures that have associated raw data
- A description of any restrictions on data availability

Sequence read data from Whole DNA and SWGA sequencing of P.falciparum genomes (as detailed in Supplementary Figure 1) is available under open-access terms from the European Nucleotide Archive (study accession ERP000190). A full list of relevant sample accessions can be found at http://www.malariagen.net/resource/32. Human genotype data used in this study has been described previously2,5 and is available under managed-access terms from the European Genome-Phenome Archive (study accession EGAS00001001311), as detailed at https://www.malariagen.net/resource/25. A dataset of the human and Pf genotypes for 3,346 severe malaria cases used in our discovery scan (Figure 1), and HbS genotypes and Pf genotypes in the larger set of 4,071 severe cases with direct HbS typing (Figure 2) is available from Zenodo under open-access terms (doi:10.5281/zenodo.4973476). A full list of data generated by this study and associated resources can be found at http://www.malariagen.net/resource/32.

Field-specific reporting

Please select the one below that is the best fit for your research. If you are not sure, read the appropriate sections before making your selection.

☒ Life sciences ☐ Behavioural & social sciences ☐ Ecological, evolutionary & environmental sciences

For a reference copy of the document with all sections, see nature.com/documents/nr-reporting-summary-flat.pdf

Life sciences study design

All studies must disclose on these points even when the disclosure is negative.

Sample size	4,171
Data exclusions	Data were excluded based on quality control of sequence read data as described in Supplementary Methods.
Replication	The main findings were replicated in additional samples from the same populations, and in uncomplicated infections from Mali as described in Main Text.
Randomization	Sample genotypes were not known at time of sample ascertainment. No formal randomisation process was followed.
Blinding	Sample genotypes were not known at time of sample ascertainment. No formal blinding process was followed.

Reporting for specific materials, systems and methods

We require information from authors about some types of materials, experimental systems and methods used in many studies. Here, indicate whether each material, system or method listed is relevant to your study. If you are not sure if a list item applies to your research, read the appropriate section before selecting a response.

Materials & experimental systems

n/a	Involved in the study	
☒	☐	Antibodies
☒	☐	Eukaryotic cell lines
☒	☐	Palaeontology and archaeology
☒	☐	Animals and other organisms
☐	☒	Human research participants
☒	☐	Clinical data
☒	☐	Dual use research of concern

Methods

n/a	Involved in the study	
☒	☐	ChIP-seq
☒	☐	Flow cytometry
☒	☐	MRI-based neuroimaging

Human research participants

Policy information about studies involving human research participants

Population characteristics	A full description of epidemiological characteristics of participants has been published previously (https://doi.org/10.1038/ng.3107)
Recruitment	Participants were recruited on attendance at district hospitals in Fajara and Banjul, The Gambia, and in Kilifi, Kenya as described previously (https://doi.org/10.1038/ng.3107)

Ethics oversight	Sample collection and design of our case-control study was approved by Oxford University Tropical Research Ethics committee (OXTREC), Oxford, United Kingdom (OXTREC 020-006). Local approving bodies were the MRC/Gambia Government Ethics Committee (SCC 1029v2 and SCC670/630) and the KEMRI Research Ethics Committee (SCC1192).

Note that full information on the approval of the study protocol must also be provided in the manuscript.
